# Color-induced changes in *Chrysanthemum morifolium*: an integrative transcriptomic and metabolomic analysis of petals and non-petals

**DOI:** 10.3389/fpls.2024.1498577

**Published:** 2024-12-20

**Authors:** Jianhong Wei, Zhaoxiang Zeng, Chengwu Song, Qing Lv, Guangya Chen, Guoyan Mo, Ling Gong, Shuna Jin, Rongzeng Huang, Bisheng Huang

**Affiliations:** ^1^ School of Pharmacy, Hubei University of Chinese Medicine, Wuhan, Hubei, China; ^2^ Hubei Shizhen Laboratory, Wuhan, Hubei, China; ^3^ Department of Pharmacy, Ezhou Central Hospital, Ezhou, Hubei, China; ^4^ Key Laboratory of Traditional Chinese Medicine Resource and Prescription, Ministry of Education, Wuhan, Hubei, China; ^5^ School of Basic Medical Sciences, Hubei University of Chinese Medicine, Wuhan, Hubei, China

**Keywords:** *Chrysanthemum morifolium*, transcriptomics, metabolomics, integrative omics, color regulation mechanism

## Abstract

*Chrysanthemum morifolium* (CM), renowned for its diverse and vibrant varieties, holds significant ornamental and medicinal value. Despite this, the core regulatory mechanisms underlying its coloration, especially in non-petal tissues (i.e., the parts of CM that do not include petals, such as the reproductive tissues, receptacle and calyx), have been insufficiently studied. In this study, we performed transcriptomic and metabolomic analyses on yellow, gold, and white CM petals, as well as non-petal tissues, to investigate the molecular processes driving color variation. A total of 90 differential metabolites were identified, with flavonoids, their derivatives, and lipids emerging as the predominant components of the metabolic profile. At the transcriptional level, 38 pathways were significantly enriched based on the expression of differential genes. The combined metabolomic and transcriptomic analyses revealed that glycerophospholipid metabolism, primarily involving lipids, served as a key regulatory pathway for both petal and non-petal parts across different tissue colors. Notably, white CM exhibited marked differences from their gold and yellow counterparts at both the metabolic and transcriptional levels. These findings offer critical insights into the molecular mechanisms governing CM coloration and provide a foundation for optimizing future breeding efforts.

## 1 Introduction

*Chrysanthemum morifolium* (CM) is a globally significant ornamental plant, extensively used for culinary and medicinal purposes, with a history spanning three thousand years (Sharma et al., 2023; Liu et al., 2024b). Evidence suggests that CM comprises a multitude of chemical constituents, including flavonoids, phenolic acids, and lipids (Chen et al., 2021; Zhou et al., 2022b). This diverse chemical composition endows CM with numerous pharmacological effects, such as anti-inflammatory, antioxidant, and chronic disease prevention properties (Li et al., 2022b, 2023c; Chen et al., 2023). Over time, and with its long history of cultivation, CM has been diversified into various varieties through hybridization with wild relatives and artificial breeding (Song et al., 2023). In addition to its medicinal value, the ornamental characteristics of CM, particularly its wide range of colors and shapes, have contributed to its commercial and cultural significance.

Significant differences exist in the composition and content of nutrients and bioactive substances among various plant varieties and their parts (Lebaka et al., 2021; Lu et al., 2021b). CM encompasses a wide range of varieties, each exhibiting distinct variations in color, shape, and functional properties (Han et al., 2019). Among these, flower color is a critical ornamental trait, significantly influencing the commercial value of CM varieties (Zhang et al., 2019; Wu et al., 2023). Understanding the mechanisms that regulate flower color in CM is helpful for advancing breeding strategies and optimizing the ornamental and medical applications of these varieties. While existing studies predominantly focus on the differences in petal color among CM varieties, the non-petal parts—such as the reproductive tissues, receptacle, and calyx—have received far less attention (Sawada et al., 2019; Zou et al., 2021). Although these non-petal parts may not exhibit visible differences across various CM colors, significant variations could exist at the molecular or metabolic level (Zhou et al., 2022a). Given that both petal and non-petal parts contribute to the ornamental and medicinal uses of CM, expanding research beyond petals offer helpful insights into the broader utility of this species. Thus, further investigation into the non-petal parts is warranted to uncover potential differences and their implications.

The advent of high-throughput technologies, including genomics, transcriptomics, and metabolomics, has greatly advanced the exploration of the intricate molecular foundations of plant biology (Guo et al., 2021; Tsugawa et al., 2021; Sun et al., 2022). These technologies have become particularly valuable in understanding the genetic and metabolic basis of traits such as flower color in CM. Metabolic profiling serves as a direct link between plant phenotypes and genotypes, identifying stage-specific metabolites and revealing the metabolic mechanisms underlying a wide variety of traits (Shen et al., 2023). Recent advancements in omics technologies have led to the increasing integration of metabolomics with other disciplines, such as transcriptomics. This synergy facilitates a systematic understanding of complex plant biological networks and fosters a more comprehensive biological knowledge base (Liu et al., 2024a; Yang et al., 2024). Investigating the alterations in plant genes and metabolites will help elucidate the molecular mechanisms underlying the non-petal and petal parts of CM exhibiting various colors.

To investigate the distinct characteristics of non-petal and petal parts in CM of varying colors and uncover potential regulatory mechanisms, white, yellow, and gold CM were subjected to transcriptomic and metabolomic analyses. Metabolomic profiling, conducted using ultra-high pressure liquid chromatography coupled with quadrupole time-of-flight mass spectrometry (UPLC-QTOF-MS/MS), revealed substantial differences between the non-petal and petal components of these differently colored CM. To further elucidate the molecular basis of coloration, RNA sequencing was performed. By integrating transcriptomic and metabolomic data, the study aimed to identify key regulatory mechanisms underlying the color-dependent differences in non-petal and petal parts. This research provides new insights into the genetic basis of CM, supporting efforts to diversify CM varieties through informed breeding strategies.

## 2 Materials and methods

### 2.1 Materials and reagents

**Figure 1:**
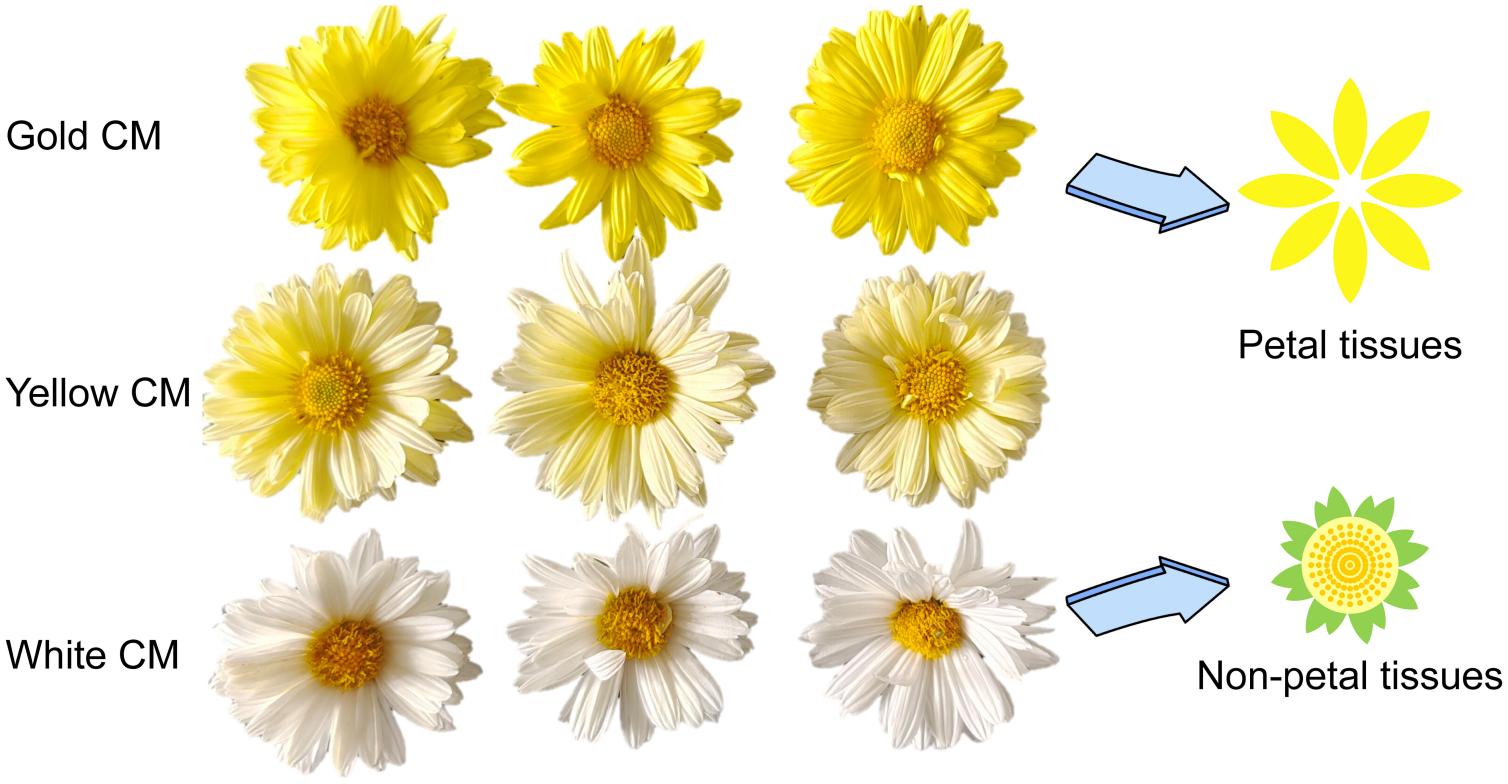
*Chrysanthemum morifolium* (CM) from three different colors-gold, yellow, and white. Non-petal tissues of CM included the reproductive tissues, receptacle, and calyx, excluding the petals.

The CM samples gathered from the medicinal botanical garden at Hubei University of Chinese Medicine were identified and authenticated by Professor Gong Ling of Hubei University of Chinese Medicine. Fresh CM samples were immediately imaged after collection and separated into petal and non-petal tissues for subsequent analysis, as shown in **Figure 1**. Non-petal samples were collected from white, yellow, and gold CM, and were designated as groups WCNP, YCNP, and GCNP, respectively. The petal samples from these colors were designated as groups WCP, YCP, and GCP. Fresh samples were stored at -80°C prior to metabolomics analysis and RNA extraction. High-performance liquid chromatography-grade acetonitrile and methanol were acquired from Merck Chemicals, Darmstadt, Germany.

### 2.2 Sample extraction for untargeted metabolomic analysis

The samples were first freeze-dried to a constant weight and then ground into a fine, uniform powder. To begin the extraction process, 100 mg of the powdered sample was weighed and extracted with 3 mL of an 80% methanol solution using ultrasonic extraction for 10 minutes. This extraction step was repeated twice: first with 3 mL of a 50% methanol solution and then with 3 mL of a 95% methanol solution. The three resulting extracts were combined, and the mixture was centrifuged at 12,000 rpm for 10 minutes. After centrifugation, the supernatant was carefully filtered and prepared for further analysis. Curcumin was employed as an internal standard in the samples at a final concentration of 200 ng mL^-1^. After filtration, the quality control (QC) sample was prepared by combining equal aliquots from all individual samples.

### 2.3 UPLC-QTOF-MS/MS-based untargeted metabolomic analysis

For the analysis of the extracts, an ACQUITY UPLC H-Class system from Waters (Milford, MA, USA) was utilized, featuring a Waters ACQUITY UPLC BEH C18 column (2.1 × 100 mm, 1.7 μm). The injection volume was 2.0 μL, the flow rate was maintained at 0.3 mL min^-1^, and the column temperature was set to 40°C. The mobile phase consisted of two solvents: mobile phase A, a solution of water and formic acid in a 1000:1 (v/v) ratio, and mobile phase B, acetonitrile. The chromatographic gradient was programmed as follows: 0 min, 5% (B); 12 min, 35% (B); 18 min, 80% (B); 22 min, 95% (B); 25 min, 95% (B); 26 min, 5% (B); and 30 min, 95% (A) and 5% (B).

Mass spectrometry analysis was carried out using a Waters Xevo G2-XS QTOF system equipped with an electrospray ionization source. The parameters were set according to our previously published reports (Zeng et al., 2023, 2024). The optimal parameters were as follows: desolvation temperature of 500°C, cone voltage of 20 V, capillary voltage of 3 KV, source temperature of 100°C, desolvation gas flow rate of 1000 L h^-1^, collision energy range of 30 to 40 eV, and cone gas flow rate of 50 L h^-1^. The mass range for full scans was set from *m/z* 50 to 1500 Da, with a scan duration of 1.0 s. Data acquisition was conducted in MS^E^ mode, employing both positive and negative ion electrospray modes. Metabolomics analysis predominantly utilized positive ion mode, while structural determination of metabolites was achieved with both ion modes.

### 2.4 RNA extraction and Illumina sequencing

RNA extraction and Illumina sequencing of the samples were primarily conducted by MetWare Biotechnology Co., Ltd. (Wuhan, Hubei, China). Total RNA was extracted from plant samples using ethanol precipitation and the CTAB-PBIOZOL method, then dissolved in DEPC-treated water. RNA integrity and concentration were assessed using a Qubit fluorescence quantifier and Qsep400 biofragment analyzer (Bioptic Inc., Taiwan, China). PolyA-tailed mRNAs were enriched with Oligo (dT) magnetic beads, fragmented, and reverse-transcribed into first-strand cDNA using random hexamer primers. Strand-specific second-strand cDNA synthesis was performed with dUTPs to ensure strand specificity, followed by end repair, A-tailing, and sequencing adapter ligation. The cDNA was size-selected (250-350 bp), PCR-amplified, purified, and quantified using Qubit 4.0 (Thermo Fisher Scientific, Massachusetts, USA) and Q-PCR (Bio-rad, California, USA). Libraries were pooled based on effective concentrations and sequenced on an Illumina platform, generating 150 bp paired-end reads via sequencing-by-synthesis. Raw sequencing data were filtered with fastp, and clean reads were assembled using Trinity. Corset was then used to remove redundant isoforms from the assembled transcripts. CDS prediction was conducted with TransDecoder, and gene function annotation was performed using DIAMOND and HMMER across databases. The fragments per kilobase of transcript per million mapped reads (FPKM) for each gene were subsequently calculated based on the gene length and the number of reads mapped to it. Transcript expression levels were quantified using RSEM, with differential expression analysis conducted using DESeq2 and edgeR, followed by Kyoto Encyclopedia of Genes and Genomes (KEGG) enrichment analyses.

### 2.5 Data analysis

The raw data from UPLC-QTOF-MS/MS were acquired using MassLynx v4.1 (Waters, Milford, MA, USA). Following data acquisition, the open-access software MS-DIAL (Version 4.9) was utilized for comprehensive data processing, encompassing peak detection, alignment, spectral deconvolution, identification, and normalization (Tsugawa et al., 2015). The retention time for the collected peaks was set between 1 and 30 minutes. Peaks with a minimum height of 3000 and *m/z* values ranging from 100 to 1500 Da were selectively retained, guided by the expected component range, while the *m/z* value for fragment ions was set between 50 and 1500 Da. Mass tolerance parameters were established at 0.015 Da for MS and 0.02 Da for MS/MS. To determine the elemental composition of the peaks, common positive ion adducts such as [M+H]^+^, [M+Na]^+^, [M+K]^+^, [M+H-H_2_O]^+^, and [2M+H]^+^ were employed. In negative ion mode, adducts like [M-H]^-^, [M+HCOOH-H]^-^, and [M-H_2_O-H]^-^ were used to assist in adduct correction alongside the positive ion adducts. Feature alignment across all samples was performed with a retention time tolerance of 0.1 and an MS tolerance of 0.015. Furthermore, the deconvolution value and MS/MS abundance cutoff were set at 0.6 and 100, respectively, to facilitate peak deconvolution. The response values of the peaks were normalized using internal standards and the LOWESS method. Finally, to ensure that the screened metabolites accurately represented each group, peaks were required to be present in at least one group, with every sample within that group exhibiting the corresponding peak.

In the search for characteristic differentially accumulated metabolites (DAMs) between two groups, we utilized variable importance in projection (VIP) values exceeding 1.25 and fold change (FC) thresholds greater than 1.5 or less than 0.67 to identify potential distinctive peaks. In the transcriptome analysis, to ensure the genes were both representative and meaningful, we applied the following criteria to eliminate false positives: (1) the average FPKM value of the gene in at least one group exceeds 1; (2) the gene’s detection rate across all samples is greater than 0.167; (3) the gene’s detection rate in any individual group is above 0.667. For the differentially expressed genes (DEGs) identified in the comparison between the two groups, the initial requirement was that genes had an FPKM > 1 in at least one group and were detected in at least two samples within any group. Subsequently, based on FPKM quantitative data, the fold change (FC) for the DEGs had to exceed 2 or fall below 0.5, with a *q*-value between the two groups required to be < 0.05. In the co-enrichment analysis, to identify pathways that represent both metabolomics and transcriptomics, we selected pathways containing at least two DAMs and conducted a comprehensive analysis of the DEGs upstream and downstream of the DAMs within these pathways.

The SIMCA 14.1 software (Umetrics AB, Umeå, Sweden) was employed for principal component analysis (PCA) and orthogonal partial least squares-discriminant analysis (OPLS-DA). Data visualization was carried out using Origin 2021 (OriginLab Corporation, Northampton, MA, USA) and R version 4.3.2 (R Foundation for Statistical Computing, Vienna, Austria). Statistical analyses were performed with SPSS version 26.0 (SPSS, Inc., Chicago, IL, USA). The significance of DAMs was assessed with a *P*-value < 0.05 using the Mann-Whitney *U* test, while DEGs were evaluated with a *q*-value < 0.05 using the Benjamini-Hochberg test. Qualitative analysis of DAMs was conducted by comparing fragment ions using literature references (Chen et al., 2017), MS-FINDER version 3.60 (Tsugawa et al., 2016), and public databases such as the Human Metabolome Database (https://www.hmdb.ca) and MassBank (https://massbank.eu/MassBank/). Quantitative determination of metabolites was achieved using Quanlynx software version 4.1 (Waters, Milford, MA, USA). For the co-enrichment analysis of metabolomics and transcriptomics, metabolic pathways of DAMs and DEGs were primarily constructed with reference to KEGG.

## 3 Results

### 3.1 Untargeted metabolomic analysis

**Figure 2:**
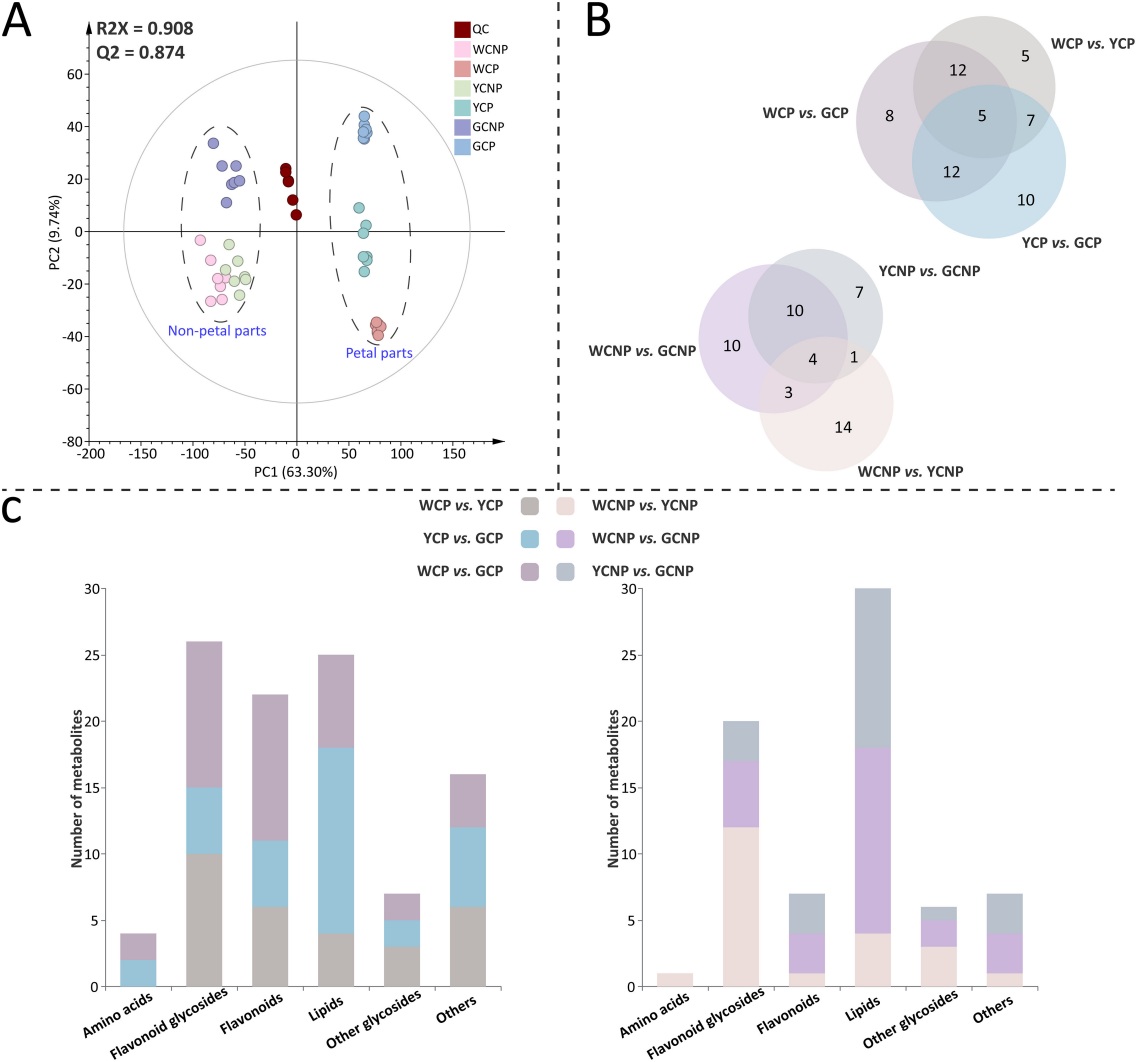
**(A)** Principal component analysis of metabolomic data from petals and non-petals of *Chrysanthemum morifolium* (CM) in three distinct colors. **(B)** Venn diagram of differentially accumulated metabolites across comparative groups. **(C)** Primary chemical classification of differentially accumulated metabolites in various comparisons. Non-petal samples from white, yellow, and gold CM were classified as WCNP, YCNP, and GCNP, respectively, while petal samples from these colors were designated as WCP, YCP, and GCP. “QC” represented the quality control group prepared from all samples.

In this study, a total of 6704 features were acquired for all samples using MS-DIAL based on UPLC-QTOF-MS/MS. As shown in **Figure 2A**, the PCA model was applied for unsupervised analysis of all samples, including quality controls. The first two principal components accounted for 63.30% and 9.74% of the variance among the samples, respectively. The results revealed clear intra-group clustering, demonstrating strong reproducibility within each sample group. Additionally, the WCNP and YCNP groups displayed a higher degree of global similarity to each other. The clustering of the QC sample further confirmed the robust stability of instrument throughout the analysis.

Furthermore, supervised OPLS-DA was employed to identify DAMs between the two groups in both petal or non-petal areas (see **Supplementary Figures S1** and **S2**). The scatter plots derived from OPLS-DA revealed clear segregation between each pair of groups. All pairwise comparisons yielded R2Y and Q2 scores consistently exceeding 0.9. Additionally, the results of the 999 permutation tests showed that the blue regression line of Q2 intersected the vertical axis below zero, confirming the absence of overfitting in the original OPLS-DA model. Ultimately, 90 DAMs (*P* < 0.05) were characterized, with a higher number identified in the petals than in the non-petal areas. The qualitative information of these metabolites, including retention time, actual *m/z* mass of the quasi-molecular ion peak, tentative identification, molecular formula, secondary fragments, and classification details, was provided in **Supplementary Table S1**. The FC and VIP values of DAMs across different comparison groups were listed in **Supplementary Table S2**. When comparing various petal groups, WCP *vs.* YCP had the fewest number of DAMs, while WCP *vs.* GCP had the most (**Figure 2B**). Meanwhile, WCNP *vs.* GCNP had the highest number of DAMs among the various non-petal groups. As illustrated in **Figure 2C**, the identified DAMs were mainly categorized into 6 categories: amino acids, flavonoid glycosides, flavonoids, lipids, other glycosides, and others. Among these, flavonoid glycosides, lipids, and flavonoids were predominantly classified in the petal parts, whereas flavonoid glycosides and lipids were primarily classified in the non-petal parts.

### 3.2 DAMs in CMs with different colors

**Figure 3:**
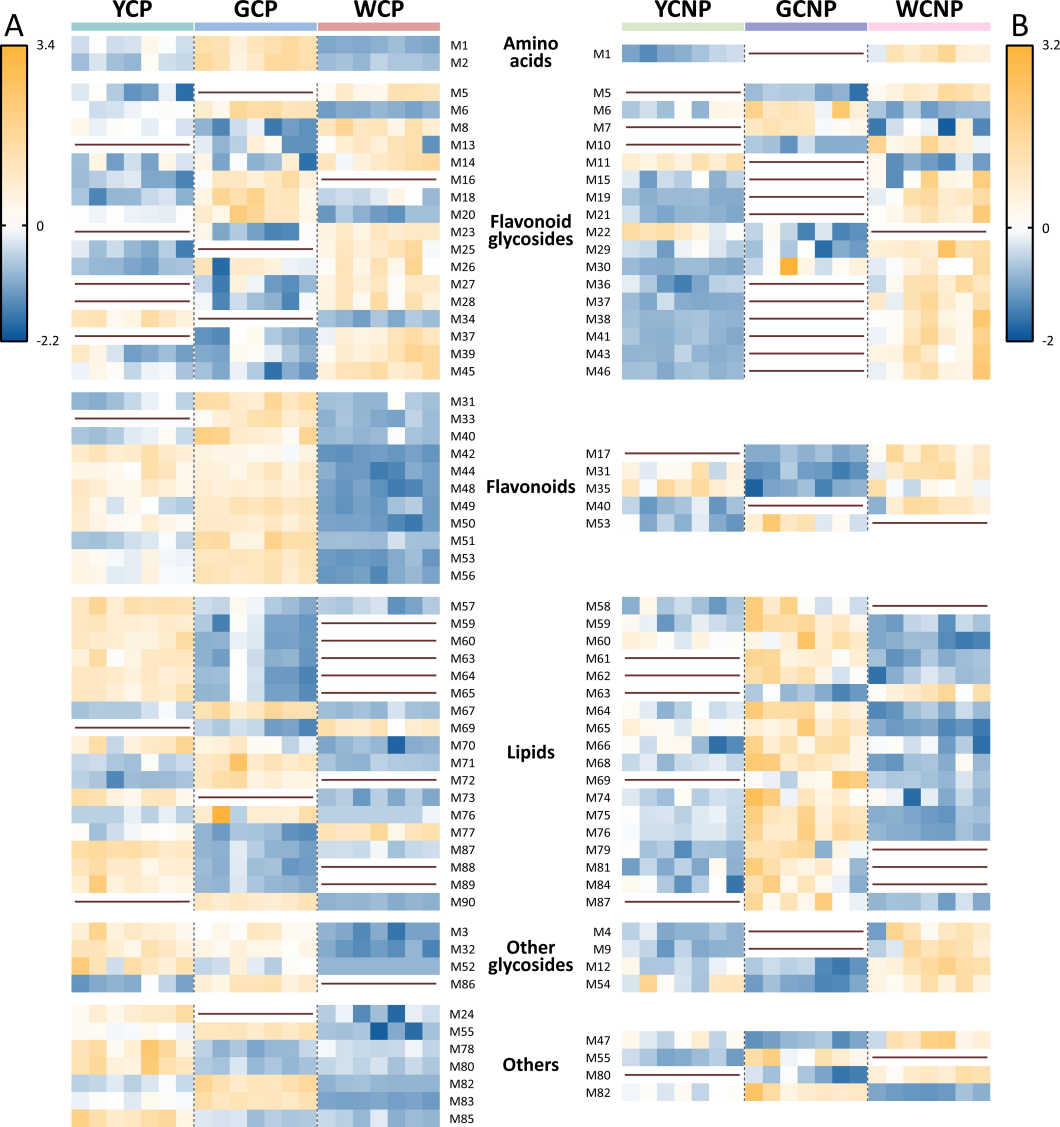
Heatmaps of differentially accumulated metabolites in petals **(A)** and non-petals **(B)** of *Chrysanthemum morifolium* (CM) across various colors. Non-petal samples from white, yellow, and gold CM were classified as WCNP, YCNP, and GCNP, respectively, while petal samples from these colors were designated as WCP, YCP, and GCP. The brown horizontal line in the figure indicated that the compound was not differentially accumulated metabolite between this group and the other groups.

To elucidate variations in the expression levels of these DAMs across different morphologies of CM, we conducted a relative quantitative analysis of the corresponding groups with the ratio of peak area between DAMs and internal standards, as shown in **Figure 3**. In the petals of CM, the following metabolite changes were observed: 8 up-regulated and 21 down-regulated in the WCP *vs.* YCP, 14 up-regulated and 20 down-regulated in YCP *vs.* GCP, and 12 up-regulated and 25 down-regulated in WCP *vs.* GCP. Similarly, in the non-petals of CM, 16 up-regulated and 6 down-regulated metabolites were identified in the WCNP *vs.* YCNP, 5 up-regulated and 17 down-regulated in YCNP *vs.* GCNP, and 11 up-regulated and 16 down-regulated in WCNP *vs.* GCNP.

As highlighted in **Figure 3**, most flavonoid glycosides showed higher concentrations in white CM, both in non-petal and petal parts. However, within the flavonoids, the petal part of white CM exhibited lower contents, while the non-petal part showed higher contents. Additionally, most lipids within DAMs were found at lower concentrations in both WCP and WCNP compared to the other two groups. Conversely, in the petals, most lipids were more abundant in YCP, whereas in the non-petal parts, they were predominantly higher in GCNP.

### 3.3 Transcriptomic analysis

**Figure 4:**
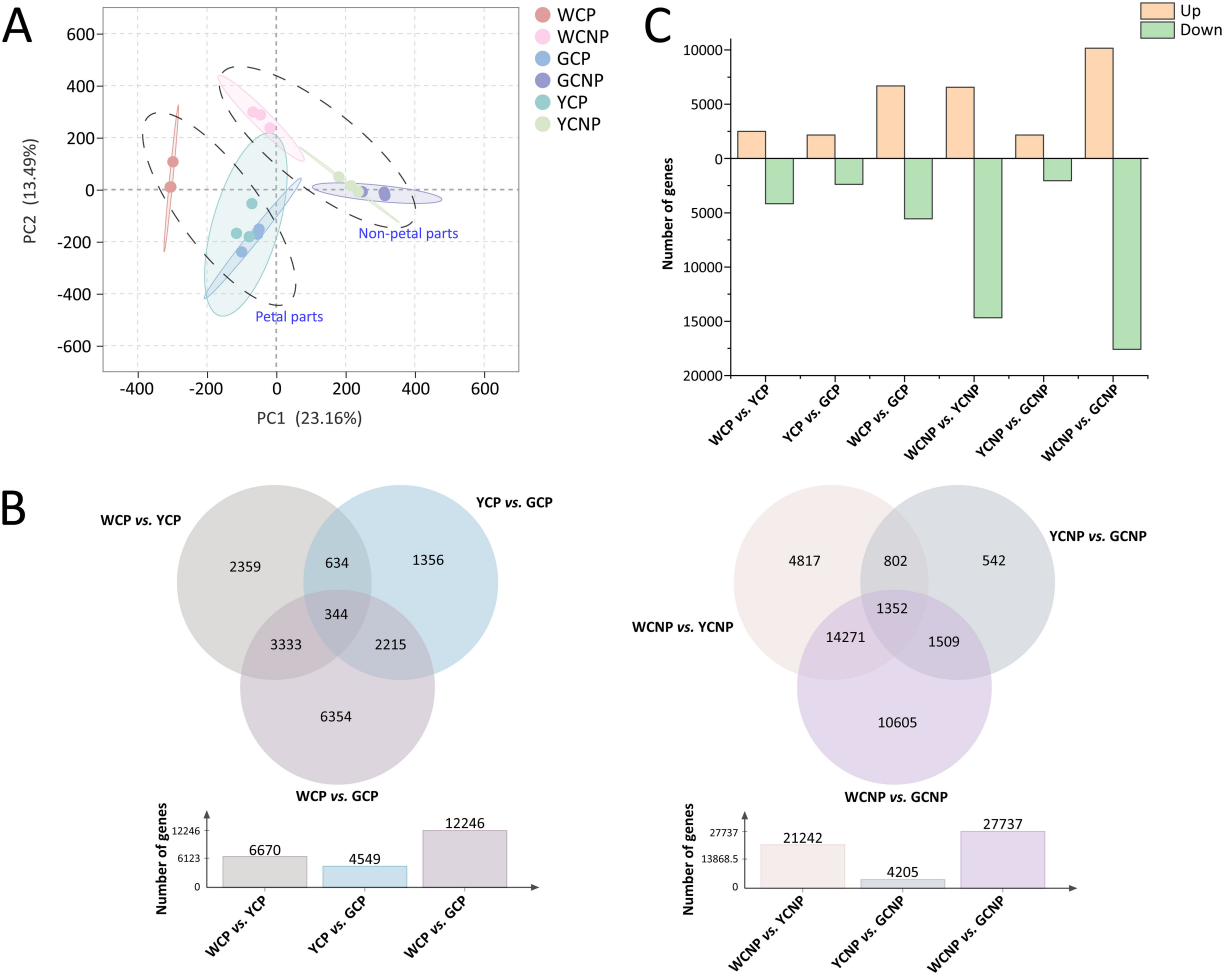
**(A)** Principal component analysis of transcription data from petals and non-petals of *Chrysanthemum morifolium* (CM) in three distinct colors. **(B)** Venn diagram of differentially expressed genes across comparative groups. **(C)** Statistics on the variation trends of differentially expressed genes. Non-petal samples from white, yellow, and gold CM were classified as WCNP, YCNP, and GCNP, respectively, while petal samples from these colors were designated as WCP, YCP, and GCP.

To gain a more comprehensive understanding of the non-petal and petal parts across different phenotypes, transcriptomic sequencing and variance analysis of DEGs were employed to investigate the underlying molecular mechanisms. After data acquisition and transcript assembly, a total of 231670 raw sequencing reads were retained. To reduce false positives while preserving valid data, 181404 reads were selected for further transcriptomic analysis. The PCA results revealed that both yellow and gold CM showed similar trends in non-petal and petal parts compared to white CM (**Figure 4A**). Moreover, each sample displayed a distinct clustering pattern within its respective group. The first two principal components accounted for 23.16% and 13.49% of the total variance among the samples, respectively. DEGs between the two groups were identified using criteria of a FC greater than 2 or less than 0.5, and a *q*-value less than 0.05. As illustrated in **Figure 4B**, the comparisons between WCP and GCP, and WCNP and GCNP, revealed the highest number of DEGs in the petal and non-petal groups, respectively. Additionally, fewer transcriptional differences were observed between yellow and gold CM.

To further illustrate the differences between the groups, a heat map was generated from the quantitative data of DEGs, as detailed in **Supplementary Figure S3**. Statistical analysis of DEG expression trends between the two groups, presented in **Figure 4C**, revealed that most DEGs exhibited a decreasing trend, particularly in comparisons between WCNP *vs.* YCNP and WCNP *vs.* GCNP.

### 3.4 DEGs in CMs with different colors

**Figure 5:**
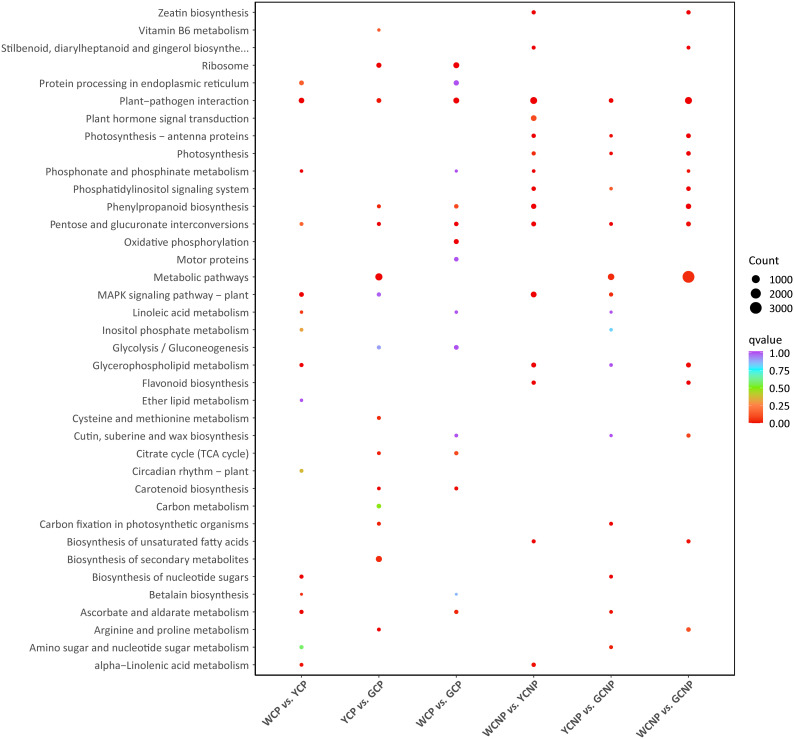
Analysis of KEGG pathway enrichment of differentially expressed genes in different comparative groups. Non-petal samples from white, yellow, and gold CM were classified as WCNP, YCNP, and GCNP, respectively, while petal samples from these colors were designated as WCP, YCP, and GCP. Only the top fifteen pathways identified through the enrichment of differentially expressed genes between the two groups were shown.

To systematically explore the biological functions of DEGs in petal and non-petal parts across different flower colors, we performed KEGG pathway enrichment analysis on the DEGs from each group separately. As shown in **Figure 5**, the top fifteen pathways enriched by DEGs in each comparison group were selected and visualized in a bubble chart.

These pathways fall into several categories, including cellular processes, environmental information processing, genetic information processing, metabolism, and organismal systems, with metabolism representing the largest portion (78.95%). Within the metabolism category, pathways related to carbohydrate and lipid metabolism were the most prevalent. Moreover, the three metabolic pathways with the highest number of DEGs were phenylpropanoid biosynthesis, pentose and glucuronate interconversions, and glycerophospholipid metabolism.

### 3.5 Integrative analysis of CMs with different colors based on metabolomics and transcriptomics

To investigate the genetic regulation of CM flower color, this study conducted a comprehensive analysis of pathways involving DEGs and DAMs, building on prior metabolomic and transcriptomic data. In order to identify pathways representing both the transcriptome and metabolome, only metabolic pathways containing at least two DAMs were retained for further analysis, with DEGs included only if located upstream or downstream of the DAMs. The KEGG codes and pathways, along with the numbers and lengths of the DEGs involved in the core pathways, were displayed in **Supplementary Table S3**.

**Figure 6:**
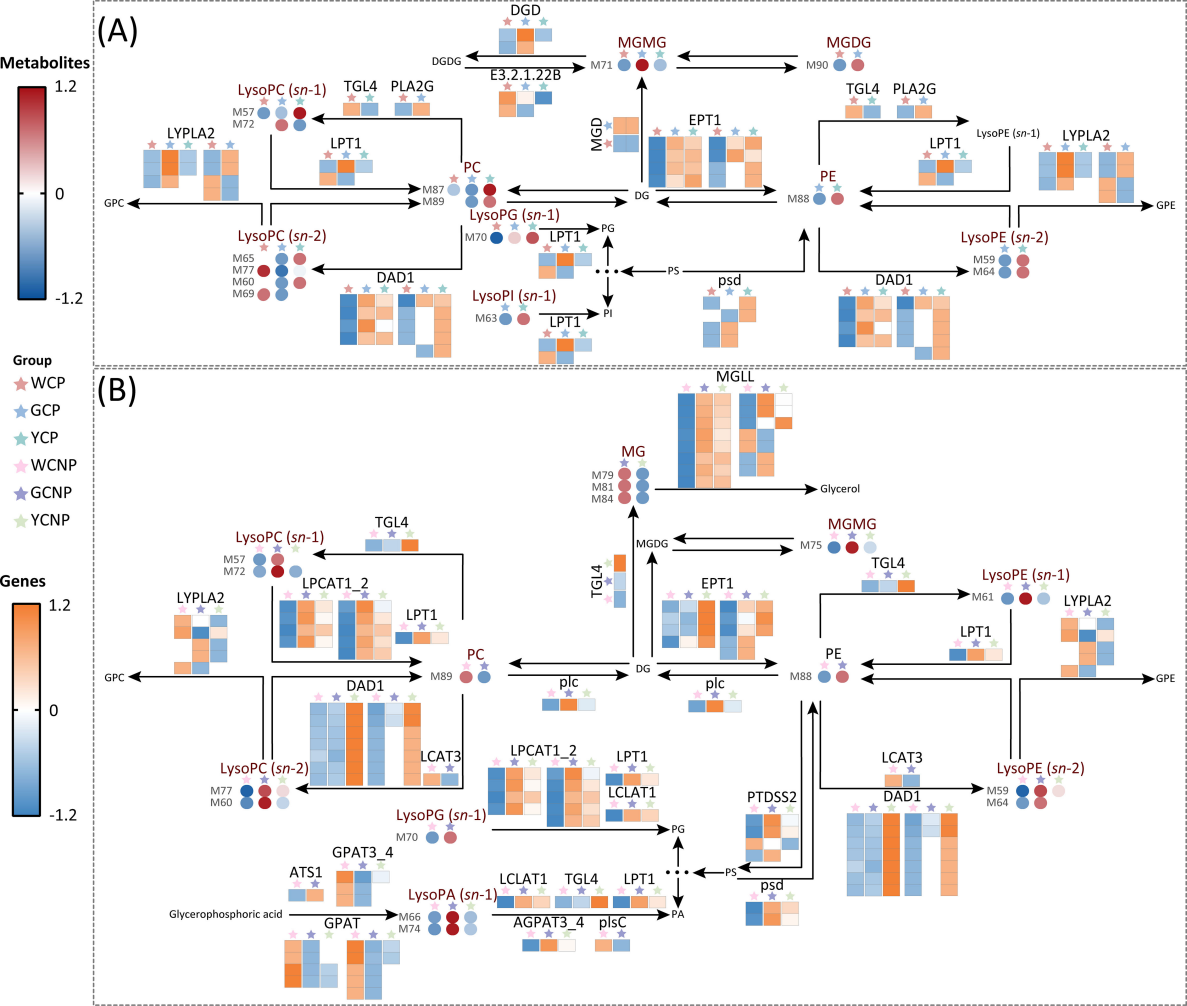
**(A)** Key metabolic pathways in the petals of *Chrysanthemum morifolium* (CM) across three distinct colors. **(B)** Key metabolic pathways in the non-petal tissues of CM from the same three colors. Non-petal samples from white, yellow, and gold CM were classified as WCNP, YCNP, and GCNP, respectively, while petal samples from these colors were designated as WCP, YCP, and GCP. The data used in the drawing were all converted by z-score normalization.

As shown in **Figure 6**, glycerophospholipid metabolism and glycerolipid metabolism, both primarily involving lipids, were the primary pathways associated with CM flower color. In petal tissues, the majority of DEGs in WCP exhibited the lowest expression compared to other groups, particularly EPT1, DAD1, MGD, and psd (**Figure 6A**). However, at the metabolite level, only MGDG, MGMG, and LysoPC (*sn*-1) showed reduced levels in WCP. Additionally, compared to YCP, a greater number of DAMs in GCP displayed a downward trend, including LysoPC (*sn*-2), PC, PE, and LysoPE (*sn*-2).

Likely, in non-petal tissues, a significant number of DEGs, including MGLL, EPT1, LPCAT1_2, and DAD1, showed reduced expression in WCNP (**Figure 6B**). Unlike the DAMs in petal tissues, the majority of DAMs in WCNPs exhibited significantly lower levels, particularly LysoPC (*sn*-1), LysoPC (*sn*-2), LysoPA (*sn*-1), and LysoPE (*sn*-2). Moreover, a substantial number of DAMs in GCP showed an upward trend compared to YCP, which contrasts with the pattern observed in the petal tissues.

## 4 Discussion

This study investigated the impact of three distinct colors (white, gold, and yellow) on both petal and non-petal tissues of CM, utilizing an integrated approach combining transcriptomics and metabolomics to uncover the molecular mechanisms and metabolic processes associated with CM coloration. At the metabolic level, 90 DAMs were identified, predominantly flavonoids, flavonoid glycosides, and lipids. Concurrently, 38 significant metabolic pathways were enriched through transcriptomics, primarily related to metabolism, which provided potential mechanistic insights into CM coloration. By integrating metabolomic and transcriptomic data, we identified two central pathways—glycerophospholipid metabolism and glycerolipid metabolism—encompassing various lipid compounds. These pathways emerged as the primary mechanisms through which flower color influenced metabolism and gene expression in CM.

As a plant with a rich cultivation history, CM has evolved a diverse range of flower colors, including white and yellow (Lu et al., 2021a). Flower color plays a pivotal role in shaping the aesthetic appeal and market demand for CM (Wan et al., 2024). Prior research on CM predominantly focused on the differential analysis of petals with notable color variations (Zou et al., 2021; Wu et al., 2023). However, our study expanded this scope, showing that varietal differences extend beyond petals to non-petal regions as well. While color variations in non-petal tissues are subtler, our results clearly demonstrated these differences.

Metabolomics, which studies metabolites as the bridge between genotype and phenotype, plays a critical role in revealing plant-environment interactions (Le et al., 2023; Shen et al., 2023). Our analysis revealed that DAMs in different CM color variants were mainly flavonoids, their derivatives, and lipids. Flavonoids and their derivatives are known functional compounds and key pharmacological constituents of CM (Chen et al., 2023; Wu et al., 2024). WCP exhibited higher levels of flavonoid glycosylation compared to YCP and GCP, with a reduction in free flavonoids and an increase in flavonoid glycosides. The decrease in flavonoids and increase in glycosylation were consistent with previous research results on white CM and yellow CM (Ouyang et al., 2022). This is likely because flavonoids contribute to vibrant coloration, offering protection against microorganisms and insects (Zhu et al., 2012; Wan et al., 2019). Lipid components, essential to plant cell composition, regulate environmental adaptability (Li et al., 2023a; Sharma et al., 2023). These distinctions were evident not only in petals but also in non-petal regions, suggesting that flower color may influence the physiological and functional properties of CM beyond the petals. Transcriptomics is a valuable tool in studying the genetic basis of plant diversity and addressing variations within species (Tyagi et al., 2022; Li et al., 2023b). Our transcriptomic analysis indicated that WCP and WCNP tissues exhibited significant differences from gold and yellow CM, with many DEGs showing a downward trend. This supported the findings of our metabolomics analysis.

The integration of transcriptomics and metabolomics provided a robust approach to identifying key pathways across plant species (Bai et al., 2021; Yuan et al., 2022). Our findings revealed that a significant number of DAMs and DEGs were co-enriched within the glycerophospholipid metabolism pathway, predominantly involving lipids. Glycerophospholipids, as essential components of cell membranes, played crucial roles in plant growth, development, and responses to environmental stimuli (Colin and Jaillais, 2020). Prior studies have noted significant differences in lipid composition among color variants, suggesting that lipid accumulation might play a key role in the coloration process (Middleton et al., 2020; Huang et al., 2024). Notably, white CM showed clear differences from yellow and gold CM. Previous studies have also highlighted significant variations in the lipid composition of CM across different varieties (Zhou et al., 2022b). Our analysis further revealed that the expression levels of most DAMs and DEGs in white CM were lower than in yellow and gold CM. This suggests that the yellow and gold coloration of CM was closely associated with the synthesis of lipid metabolites and the expression of related genes. The upregulation of genes related to glycerophospholipid metabolism enhances lipid levels and increases the plant’s resilience to environmental stress (Liu et al., 2022). Other studies have shown that environmental changes can alter glycerophospholipid composition, modulating plant responses and potentially leading to color variation (Zhou et al., 2022c; Li et al., 2024). Importantly. phospholipase A1 (DAD1) and acylglycerol lipase (MGLL) were identified as critical enzymes in mediating plant responses to both biotic and abiotic stress (Zhang et al., 2021; Zhao et al., 2024). Additionally, lysophospholipids accumulate in plants under stress conditions, such as freezing, injury, and pathogen infection (Hou et al., 2016). Since carotenoids are synthesized in plant chloroplasts and glycerophospholipid transport also occurred within these organelles, flower color might be regulated through this indirect pathway (Vishnevetsky et al., 1999; Benning, 2008). Therefore, the environmental adaptability of CM likely regulates metabolites and genes involved in glycerophospholipid metabolism, leading to color changes. Furthermore, earlier research indicated that yellow CM exhibited superior adaptability compared to white CM, further supporting our findings (Chumber and Jhanji, 2022).

As early as the Tang Dynasty in China, only yellow varieties of CM were cultivated (Song et al., 2023). Due to their high ornamental value, years of cultivation and refinement expanded their colors to include yellow, gold, white, pink, and more (Su et al., 2019). Understanding the mechanisms behind this coloration had proven highly beneficial in enhancing their economic value. If the identified DAMs and DEGs were validated through further experimental verification, the expression of plant DEGs could be modulated by altering environmental conditions, introducing exogenous stimuli, or applying advanced genetic technologies based on the results. Relevant literature has reported the modification of plant color through methods such as environmental changes, introduction of exogenous interference, and gene silencing (Li et al., 2022a; Yangyang et al., 2022; Zhou et al., 2022c). This modulation would then regulate DAM expression, leading to the desired color. This strategy also represents a key direction for us to further explore through experiments in the future. While the most apparent differences in color lay in the petals, identifying distinctions across the entire plant, including both petals and non-petal parts, provided deeper insights into the coloration process. The integration of transcriptomics and metabolomics in this study allowed us to reveal how metabolic and genetic pathways affect non-petal tissues. These findings suggested that metabolic and genetic changes in response to flower color variation were not limited to petals, where phenotypes varied more, but may also have affected non-petal tissues. For instance, metabolic pathways involved in lipid metabolism, such as glycerophospholipid and glycerolipid biosynthesis, were found to be active in both petals and non-petal tissues, indicating that flower color regulation not only had a significant impact on the metabolic processes in petal tissues, but also on non-petal tissues.

## 5 Conclusion

This study employed transcriptomic and metabolomic analyses to elucidate the underlying mechanisms influencing color both in petal and non-petal tissues of CM. Pairwise comparisons of gene expression and metabolite levels across different colors revealed that lipids, flavonoids, and their derivatives were the principal metabolites affected. The glycerophospholipid metabolism, predominantly composed of lipids, and its associated gene variations emerged as crucial factors contributing to color differences in CM. Notably, significant metabolic and genetic distinctions were observed between white CM and their yellow and gold counterparts, extending beyond the petals to the non-petal tissues. Understanding the role of glycerophospholipid metabolism in flower coloration can provide a scientific basis for developing strategies to modulate flower color through environmental or genetic interventions. Furthermore, glycerophospholipids, which play a role in plant stress response and environmental adaptability, could be leveraged to breed CM varieties with improved resilience to abiotic stresses, thus contributing to sustainable cultivation practices. This research establishes a foundation for further exploration of CM coloration pathways and provides a scientific basis for quality control, cultivation, and enhancement strategies for CM.

## Data Availability

The datasets presented in this study can be found in online repositories. The names of the repository/repositories and accession number(s) can be found below: https://www.ncbi.nlm.nih.gov/, https://www.ncbi.nlm.nih.gov/bioproject/PRJNA1162299.

## References

[B1] Bai Y. Liu H. Pan J. Zhang S. Guo Y. Xian Y.. (2021). Transcriptomics and metabolomics changes triggered by inflorescence removal in *Panax notoginseng* (burk.). Front. Plant Sci. 12. doi: 10.3389/fpls.2021.761821 PMC8636121 34868157

[B2] Benning C. (2008). A role for lipid trafficking in chloroplast biogenesis. Prog. Lipid Res. 47, 381–389. doi: 10.1016/j.plipres.2008.04.001 18440317

[B3] Chen S. Liu J. Dong G. Zhang X. Liu Y. Sun W.. (2021). Flavonoids and caffeoylquinic acids in *Chrysanthemum morifolium* Ramat flowers: A potentially rich source of bioactive compounds. Food Chem. 344, 128733. doi: 10.1016/j.foodchem.2020.128733 33280963

[B4] Chen G. Song C. Jin S. Li S. Zhang Y. Huang R.. (2017). An integrated strategy for establishment of metabolite profile of endogenous lysoglycerophospholipids by two LC-MS/MS platforms. Talanta 162, 530–539. doi: 10.1016/j.talanta.2016.10.045 27837867

[B5] Chen L. Sun J. Pan Z. Lu Y. Wang Z. Yang L.. (2023). Analysis of chemical constituents of *Chrysanthemum morifolium* extract and its effect on postprandial lipid metabolism in healthy adults. Molecules 28, 579. doi: 10.3390/molecules28020579 36677639 PMC9866508

[B6] Chumber M. Jhanji S. (2022). Morpho-physiological and biochemical characterization of chrysanthemum varieties for early flowering under heat stress. S. Afr. J. Bot. 146, 603–613. doi: 10.1016/j.sajb.2021.11.035

[B7] Colin L. A. Jaillais Y. (2020). Phospholipids across scales: Lipid patterns and plant development. Curr. Opin. Plant Biol. 53, 1–9. doi: 10.1016/j.pbi.2019.08.007 31580918

[B8] Guo J. Huang Z. Sun J. Cui X. Liu Y. (2021). Research progress and future development trends in medicinal plant transcriptomics. Front. Plant Sci. 12. doi: 10.3389/fpls.2021.691838 PMC8355584 34394145

[B9] Han A.-R. Nam B. Kim B.-R. Lee K.-C. Song B.-S. Kim S. H.. (2019). Phytochemical composition and antioxidant activities of two different color chrysanthemum flower teas. Molecules 24, 329. doi: 10.3390/molecules24020329 30658439 PMC6359479

[B10] Hou Q. Ufer G. Bartels D. (2016). Lipid signalling in plant responses to abiotic stress. Plant Cell Environ. 39, 1029–1048. doi: 10.1111/pce.12666 26510494

[B11] Huang F. Yang P. Bai S. Liu Z. Li J. Huang J.. (2024). Lipids: A noteworthy role in better tea quality. Food Chem. 431, 137071. doi: 10.1016/j.foodchem.2023.137071 37582323

[B12] Le Q. T. N. Sugi N. Yamaguchi M. Hirayama T. Kobayashi M. Suzuki Y.. (2023). Morphological and metabolomics profiling of intraspecific *Arabidopsis* hybrids in relation to biomass heterosis. Sci. Rep. 13, 9529. doi: 10.1038/s41598-023-36618-y 37308530 PMC10261038

[B13] Lebaka V. R. Wee Y.-J. Ye W. Korivi M. (2021). Nutritional composition and bioactive compounds in three different parts of mango fruit. Int. J. Environ. Res. Public Health 18, 741. doi: 10.3390/ijerph18020741 33467139 PMC7830918

[B14] Li X. Cao J. Zhao H. Jiang G. Liu J. Yu Y. (2022a). Ph5GT silencing alters flower color and flavonoids metabolome profile in petunia. Physiol. Plant 174, e13795. doi: 10.1111/ppl.13795 36193023

[B15] Li X. Li R. Wang X. Zhang X. Xiao Z. Wang H.. (2023c). Effects and mechanism of action of *Chrysanthemum morifolium* (jinsi huangju) on hyperlipidemia and non-alcoholic fatty liver disease. Eur. J. Med. Chem. 255, 115391. doi: 10.1016/j.ejmech.2023.115391 37099836

[B16] Li Y. Liu X. Su S. Yan H. Guo S. Qian D.. (2022b). Evaluation of anti-inflammatory and antioxidant effects of chrysanthemum stem and leaf extract on zebrafish inflammatory bowel disease model. Molecules 27, 2114. doi: 10.3390/molecules27072114 35408512 PMC9000279

[B17] Li S. Nakayama H. Sinha N. R. (2023b). How to utilize comparative transcriptomics to dissect morphological diversity in plants. Curr. Opin. Plant Biol. 76, 102474. doi: 10.1016/j.pbi.2023.102474 37804608

[B18] Li M. Yu A. Sun Y. Hu Q. Kang J. Chen L.. (2023a). Lipid composition remodeling and storage lipid conversion play a critical role in salt tolerance in alfalfa (*Medicago sativa* L.) leaves. Environ. Exp. Bot. 205, 105144. doi: 10.1016/j.envexpbot.2022.105144

[B19] Li T. Zheng C. Wu J. Xu W. Yan T. Liu J.. (2024). Comparative lipidomics analysis provides new insights into the metabolic basis of color formation in green cotton fiber. Plants 13, 3063. doi: 10.3390/plants13213063 39519983 PMC11548578

[B20] Liu Y. Lu C. Zhou J. Zhou F. Gui A. Chu H.. (2024b). *Chrysanthemum morifolium* as a traditional herb: A review of historical development, classification, phytochemistry, pharmacology and application. J. Ethnopharmacol. 330, 118198. doi: 10.1016/j.jep.2024.118198 38621465

[B21] Liu H. Xin W. Wang Y. Zhang D. Wang J. Zheng H.. (2022). An integrated analysis of the rice transcriptome and lipidome reveals lipid metabolism plays a central role in rice cold tolerance. BMC Plant Biol. 22, 91. doi: 10.1186/s12870-022-03468-1 35232394 PMC8889772

[B22] Liu C. Zhou G. Qin H. Guan Y. Wang T. Ni W.. (2024a). Metabolomics combined with physiology and transcriptomics reveal key metabolic pathway responses in apple plants exposure to different selenium concentrations. J. Hazard. Mater. 464, 132953. doi: 10.1016/j.jhazmat.2023.132953 37952334

[B23] Lu C. Li Y. Wang J. Qu J. Chen Y. Chen X.. (2021a). Flower color classification and correlation between color space values with pigments in potted multiflora chrysanthemum. Sci. Hortic. 283, 110082. doi: 10.1016/j.scienta.2021.110082

[B24] Lu X. Zhao C. Shi H. Liao Y. Xu F. Du H.. (2021b). Nutrients and bioactives in citrus fruits: Different citrus varieties, fruit parts, and growth stages. Crit. Rev. Food Sci. Nutr. 63, 2018–2041. doi: 10.1080/10408398.2021.1969891 34609268

[B25] Middleton R. Sinnott-Armstrong M. Ogawa Y. Jacucci G. Moyroud E. Rudall P. J.. (2020). *Viburnum tinus* fruits use lipids to produce metallic blue structural color. Curr. Biol. 30, 3804–3810. doi: 10.1016/j.cub.2020.07.005 32763166

[B26] Ouyang H. Fan Y. Wei S. Chang Y. He J. (2022). Study on the chemical profile of chrysanthemum (*Chrysanthemum morifolium*) and the evaluation of the similarities and differences between different cultivars. Chem. Biodivers. 19, e202200252. doi: 10.1002/cbdv.202200252 35831709

[B27] Sawada Y. Sato M. Okamoto M. Masuda J. Yamaki S. Tamari M.. (2019). Metabolome-based discrimination of chrysanthemum cultivars for the efficient generation of flower color variations in mutation breeding. Metabolomics 15, 118. doi: 10.1007/s11306-019-1573-7 31451959

[B28] Sharma N. Radha, Kumar M. Kumari N. Puri S. Rais N.. (2023). Phytochemicals, therapeutic benefits and applications of chrysanthemum flower: A review. Heliyon 9, e20232. doi: 10.1016/j.heliyon.2023.e20232 37860517 PMC10582400

[B29] Shen S. Zhan C. Yang C. Fernie A. R. Luo J. (2023). Metabolomics-centered mining of plant metabolic diversity and function: Past decade and future perspectives. Mol. Plant 16, 43–63. doi: 10.1016/j.molp.2022.09.007 36114669

[B30] Song A. Su J. Wang H. Zhang Z. Zhang X. Van de Peer Y.. (2023). Analyses of a chromosome-scale genome assembly reveal the origin and evolution of cultivated chrysanthemum. Nat. Commun. 14, 2021. doi: 10.1038/s41467-023-37730-3 37037808 PMC10085997

[B31] Su J. Jiang J. Zhang F. Liu Y. Ding L. Chen S.. (2019). Current achievements and future prospects in the genetic breeding of chrysanthemum: A review. Hortic. Res. 6, 109. doi: 10.1038/s41438-019-0193-8 31666962 PMC6804895

[B32] Sun Y. Shang L. Zhu Q.-H. Fan L. Guo L. (2022). Twenty years of plant genome sequencing: Achievements and challenges. Trends Plant Sci. 27, 391–401. doi: 10.1016/j.tplants.2021.10.006 34782248

[B33] Tsugawa H. Cajka T. Kind T. Ma Y. Higgins B. Ikeda K.. (2015). MS-DIAL: Data-independent MS/MS deconvolution for comprehensive metabolome analysis. Nat. Methods 12, 523–526. doi: 10.1038/nmeth.3393 25938372 PMC4449330

[B34] Tsugawa H. Kind T. Nakabayashi R. Yukihira D. Tanaka W. Cajka T.. (2016). Hydrogen rearrangement rules: Computational MS/MS fragmentation and structure elucidation using MS-FINDER software. Anal. Chem. 88, 7946–7958. doi: 10.1021/acs.analchem.6b00770 27419259 PMC7063832

[B35] Tsugawa H. Rai A. Saito K. Nakabayashi R. (2021). Metabolomics and complementary techniques to investigate the plant phytochemical cosmos. Nat. Prod. Rep. 38, 1729–1759. doi: 10.1039/d1np00014d 34668509

[B36] Tyagi P. Singh D. Mathur S. Singh A. Ranjan R. (2022). Upcoming progress of transcriptomics studies on plants: An overview. Front. Plant Sci. 13. doi: 10.3389/fpls.2022.1030890 PMC9798009 36589087

[B37] Vishnevetsky M. Ovadis M. Vainstein A. (1999). Carotenoid sequestration in plants: The role of carotenoid-associated proteins. Trends Plant Sci. 4, 232–235. doi: 10.1016/S1360-1385(99)01414-4 10366880

[B38] Wan W. Jia F. Liu Z. Sun W. Zhang X. Su J.. (2024). Quantitative evaluation and genome-wide association studies of chrysanthemum flower color. Sci. Hortic. 338, 113561. doi: 10.1016/j.scienta.2024.113561

[B39] Wan H. Yu C. Han Y. Guo X. Luo L. Pan H.. (2019). Determination of flavonoids and carotenoids and their contributions to various colors of rose cultivars (*Rosa* spp.). Front. Plant Sci. 10. doi: 10.3389/fpls.2019.00123 PMC6379320 30809238

[B40] Wu D. Wu Y. Gao R. Zhang Y. Zheng R. Fang M.. (2024). Integrated metabolomics and transcriptomics reveal the key role of flavonoids in the cold tolerance of chrysanthemum. Int. J. Mol. Sci. 25, 7589. doi: 10.3390/ijms25147589 39062834 PMC11276724

[B41] Wu D. Zhuang F. Wang J. Gao R. Zhang Q. Wang X.. (2023). Metabolomics and transcriptomics revealed a comprehensive understanding of the biochemical and genetic mechanisms underlying the color variations in chrysanthemums. Metabolites 13, 742. doi: 10.3390/metabo13060742 37367900 PMC10301146

[B42] Yang X. Chen Y. Liu W. Huang T. Yang Y. Mao Y.. (2024). Combined transcriptomics and metabolomics to analyse the response of *Cuminum cyminum* L. under pb stress. Sci. Total Environ. 923, 171497. doi: 10.1016/j.scitotenv.2024.171497 38453091

[B43] Yangyang Y. Qin L. Kun Y. Xiaoyi W. Pei X. (2022). Transcriptomic and metabolomic analyses reveal how girdling promotes leaf color expression in *Acer rubrum* L. BMC Plant Biol. 22, 498. doi: 10.1186/s12870-022-03776-6 36280828 PMC9590220

[B44] Yuan Z. Dong F. Pang Z. Fallah N. Zhou Y. Li Z.. (2022). Integrated metabolomics and transcriptome analyses unveil pathways involved in sugar content and rind color of two sugarcane varieties. Front. Plant Sci. 13. doi: 10.3389/fpls.2022.921536 PMC9244704 35783968

[B45] Zeng Z. Jin S. Xiang X. Yuan H. Jin Y. Shi Q.. (2023). Dynamical changes of tea metabolites fermented by *Aspergillus cristatus*, *Aspergillus neoniger* and mixed fungi: A temporal clustering strategy for untargeted metabolomics. Food Res. Int. 170, 112992. doi: 10.1016/j.foodres.2023.112992 37316065

[B46] Zeng Z. Song C. Hu X. Zhu X. Li Y. Ren J.. (2024). Constituent-taste relationship of Kuding tea fermented by *Aspergillus neoniger* and *Aspergillus cristatus*: Unveiling taste characteristics through untargeted metabolomics. Food Bioscience 62, 105027. doi: 10.1016/j.fbio.2024.105027

[B47] Zhang X. Xu Z. Yu X. Zhao L. Zhao M. Han X.. (2019). Identification of Two Novel R2R3-MYB Transcription factors, PsMYB114L and PsMYB12L, Related to Anthocyanin Biosynthesis in Paeonia suffruticosa. Int. J. Mol. Sci. 20, 1055. doi: 10.3390/ijms20051055 30823465 PMC6429501

[B48] Zhang H. Zhang Y. Xu N. Rui C. Fan Y. Wang J.. (2021). Genome-wide expression analysis of phospholipase A1 (PLA1) gene family suggests phospholipase A1-32 gene responding to abiotic stresses in cotton. Int. J. Biol. Macromol. 192, 1058–1074. doi: 10.1016/j.ijbiomac.2021.10.038 34656543

[B49] Zhao Y. Li S. Wu J. Liu H. Wang P. Xu L. (2024). Insights into membrane lipids modification in barley leaves as an adaptation mechanism to cold stress. Plant Growth Regul. 103, 369–388. doi: 10.1007/s10725-023-01114-w

[B50] Zhou L. Cai Y. Yang L. Zou Z. Zhu J. Zhang Y. (2022a). Comparative metabolomics analysis of stigmas and petals in Chinese saffron (*Crocus sativus*) by widely targeted metabolomics. Plants 11, 2427. doi: 10.3390/plants11182427 36145828 PMC9502368

[B51] Zhou Z. Chen M. Wu Q. Zeng W. Chen Z. Sun W. (2022c). Combined analysis of lipidomics and transcriptomics revealed the key pathways and genes of lipids in light-sensitive albino tea plant (*Camellia sinensis* cv. *Baijiguan*). Front. Plant Sci. 13. doi: 10.3389/fpls.2022.1035119 PMC9623167 36330254

[B52] Zhou L. Ma Y. Yao J. Zhang M. Fu H. Yang J.. (2022b). Discrimination of chrysanthemum varieties using lipidomics based on UHPLC-HR-AM/MS/MS. J. Sci. Food Agric. 103, 837–845. doi: 10.1002/jsfa.12195 36044335

[B53] Zhu M. Zheng X. Shu Q. Li H. Zhong P. Zhang H.. (2012). Relationship between the composition of flavonoids and flower colors variation in tropical water lily (nymphaea) cultivars. PloS One 7, e34335. doi: 10.1371/journal.pone.0034335 22485167 PMC3317528

[B54] Zou Q. Wang T. Guo Q. Yang F. Chen J. Zhang W. (2021). Combined metabolomic and transcriptomic analysis reveals redirection of the phenylpropanoid metabolic flux in different colored medicinal *Chrysanthemum morifolium* . Ind. Crops Prod. 164, 113343. doi: 10.1016/j.indcrop.2021.113343

