# Supplementary material for: Color-induced changes in Chrysanthemum morifolium: an integrative transcriptomic and metabolomic analysis of petals and non-petals
Source: Front Plant Sci. 2024 Dec 20;15:1498577. doi: 10.3389/fpls.2024.1498577 (PMC11695349; doi:10.3389/fpls.2024.1498577)
Supplement: Supplementary file 1 [file DataSheet1.docx]

***Supplementary Material***

**Color-Induced Changes in *Chrysanthemum morifolium*: An Integrative Transcriptomic and Metabolomic Analysis of Petals and Non-Petals**

**Contents**

**Figure S1**. The score plot of the OPLS-DA model and the permutation test plot with 999 replications for WCP *vs.* GCP, WCP *vs.* YCP, and YCP *vs.* GCP. Petal samples were obtained from white, yellow, and gold *Chrysanthemum morifolium*, categorized as WCP, YCP, and GCP, respectively.

**Figure S2**. The score plot of the OPLS-DA model and the permutation test plot with 999 replications for WCNP *vs.* GCNP, WCNP *vs.* YCNP, and YCNP *vs.* GCNP. Non- Petal samples were obtained from white, yellow, and gold *Chrysanthemum morifolium*, categorized as WCNP, YCNP, and GCNP, respectively.

**Figure S3**. Heatmaps of differentially expressed genes in *Chrysanthemum morifolium* (CM) for YCNP *vs.* GCNP (A), WCNP *vs.* GCNP (B), WCNP *vs.* YCNP (C), YCP *vs.* GCP (D), WCP *vs.* GCP (E), and WCP *vs.* YCP (F). Non-petal samples from white, yellow, and gold CM were classified as WCNP, YCNP, and GCNP, respectively, while petal samples from these colors were designated as WCP, YCP, and GCP.

**Table S1**. Identification information of differential metabolites in UPLC-QTOF-MS/MS analysis.

**Table S2**. Variable importance in projection values and fold changes of differential metabolites.

**Table S3**. Annotation of differentially expressed genes in key pathways.


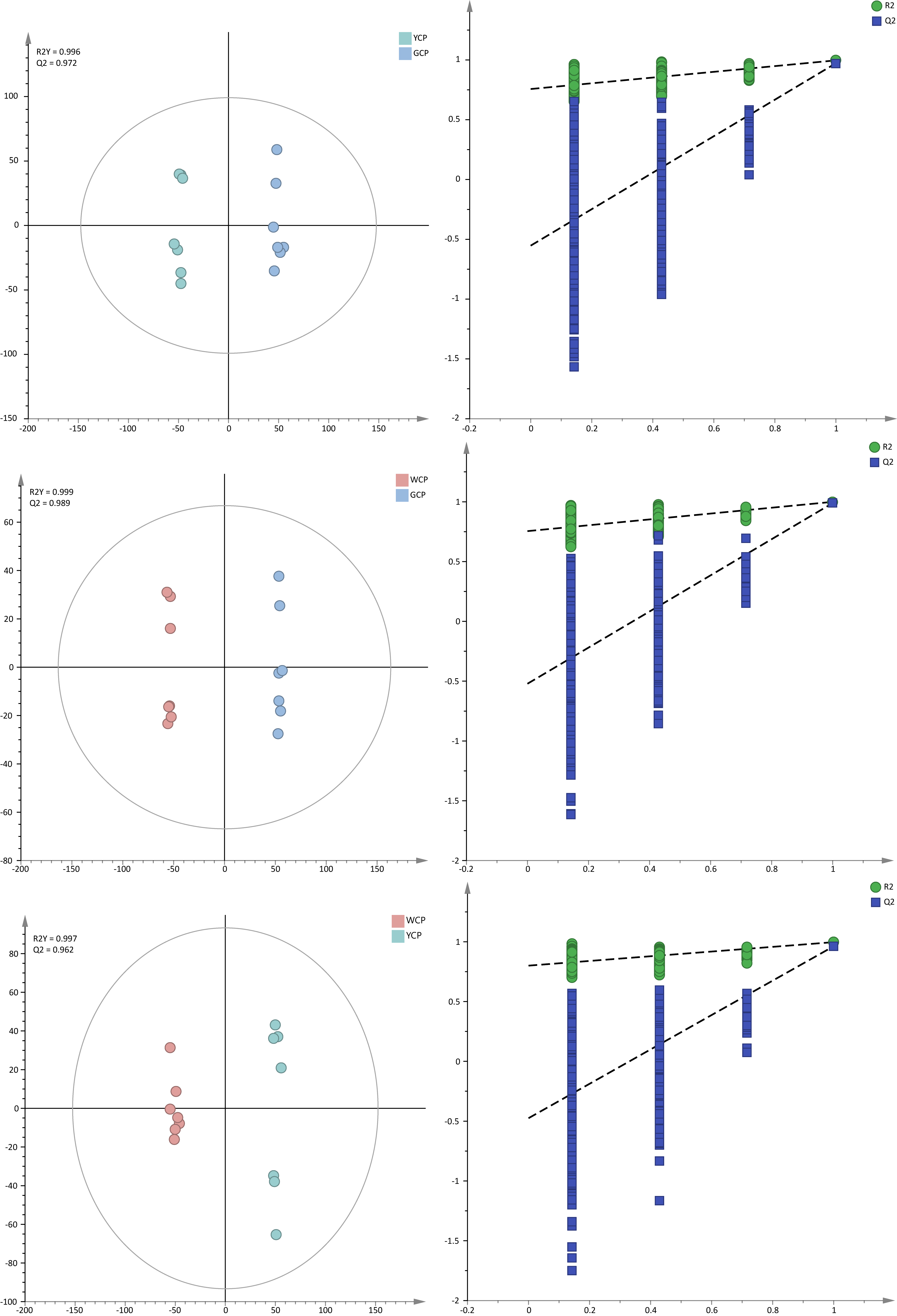


**Figure S1**. The score plot of the OPLS-DA model and the permutation test plot with 999 replications for WCP *vs.* GCP, WCP *vs.* YCP, and YCP *vs.* GCP. Petal samples were obtained from white, yellow, and gold *Chrysanthemum morifolium*, categorized as WCP, YCP, and GCP, respectively.


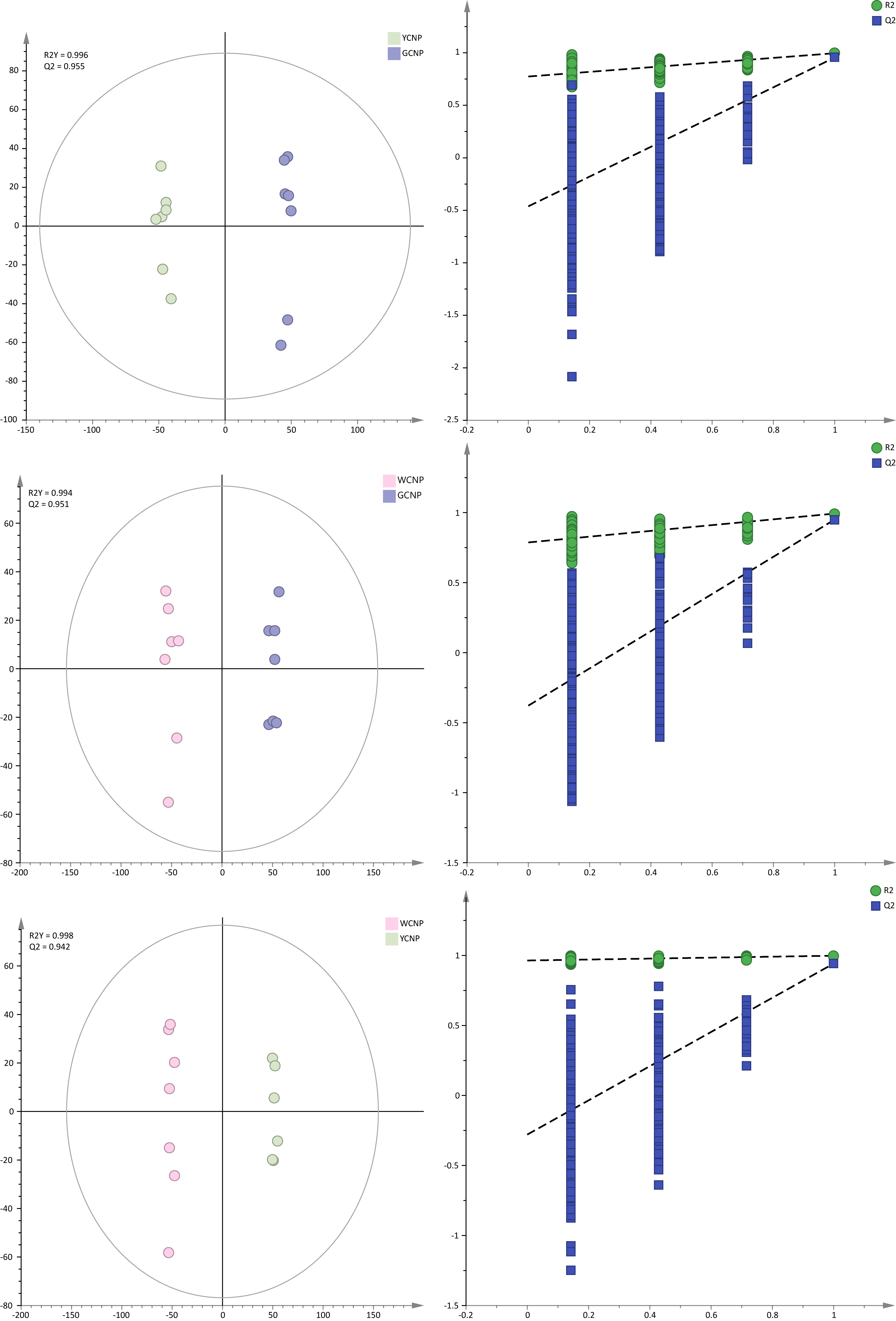


**Figure S2**. The score plot of the OPLS-DA model and the permutation test plot with 999 replications for WCNP *vs.* GCNP, WCNP *vs.* YCNP, and YCNP *vs.* GCNP. Non- Petal samples were obtained from white, yellow, and gold *Chrysanthemum morifolium*, categorized as WCNP, YCNP, and GCNP, respectively.


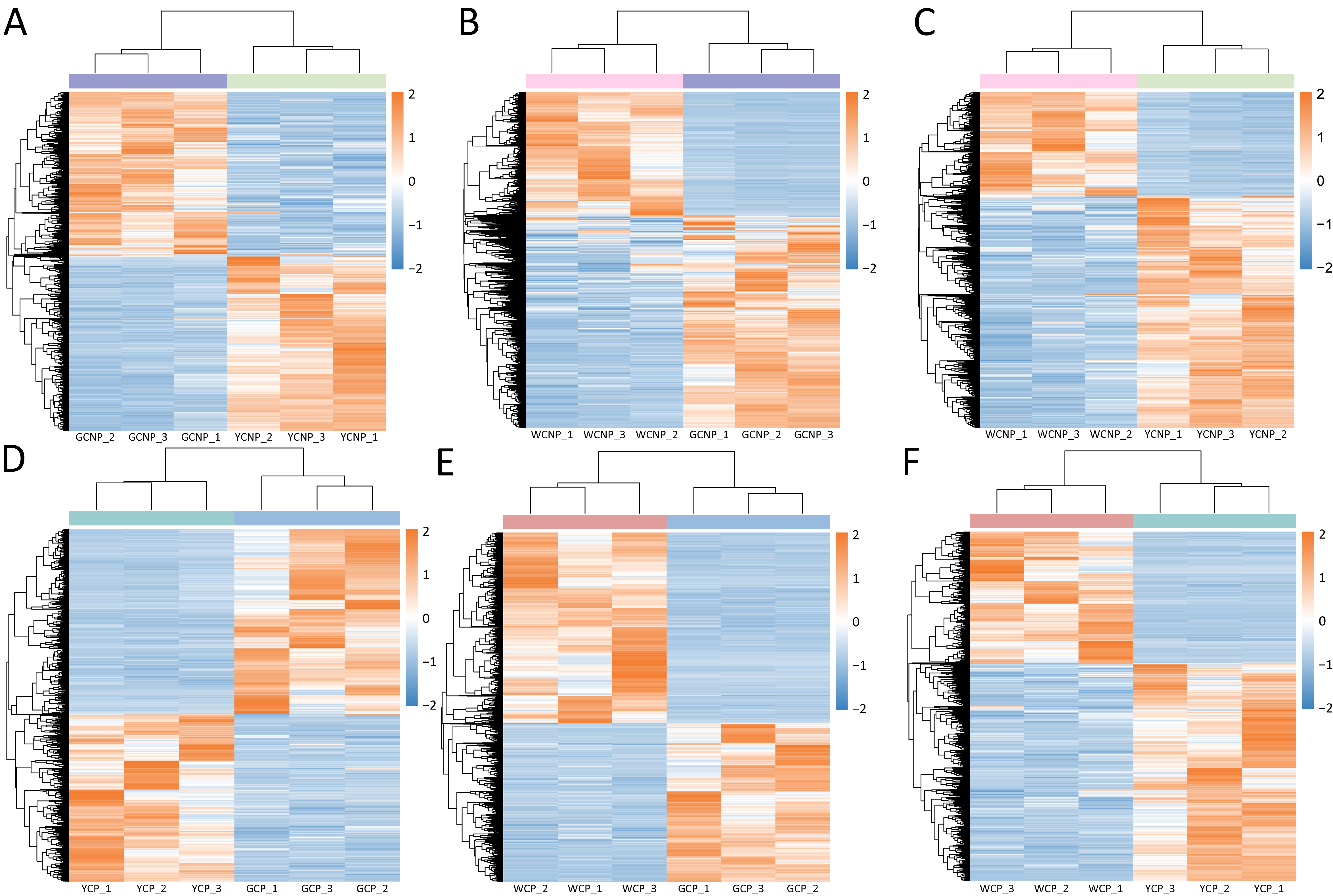


**Figure S3**. Heatmaps of differentially expressed genes in *Chrysanthemum morifolium* (CM) for YCNP *vs.* GCNP (A), WCNP *vs.* GCNP (B), WCNP *vs.* YCNP (C), YCP *vs.* GCP (D), WCP *vs.* GCP (E), and WCP *vs.* YCP (F). Non-petal samples from white, yellow, and gold CM were classified as WCNP, YCNP, and GCNP, respectively, while petal samples from these colors were designated as WCP, YCP, and GCP.

**Table S1**. Identification information of differential metabolites in UPLC-QTOF-MS/MS analysis.

| No. | RT (min) | Mass (*m/z*) | Adduct type | Mass error (ppm) | Tentative identification | Formula | MS2 fragments | Class Ⅰ | Class Ⅱ |
| --- | --- | --- | --- | --- | --- | --- | --- | --- | --- |
| M1 | 1.92 | 166.0867 | [M + H]^+^ | -0.60 | Phenylalanine | C9H11NO2 | 91.05, 103.05, 120.08 | Amino acids | Phenylalanine and derivatives |
| M2 | 3.04 | 205.0974 | [M + H]^+^ | -1.46 | Tryptophan | C11H12N2O2 | 115.05, 130.06, 143.07 | Amino acids | Tryptophan and derivatives |
| M3 | 3.31 | 517.1554 | [M + H]^+^ | -0.58 | 5-Hydroxy-2-Methylchromone-7-O-Rutinoside | C22H28O14 | 163.04, 347.08 | Other glycosides | Chromone glycosides |
| M4 | 4.00 | 509.1274 | [M + Na]^+^ | 0.59 | Hymexelsin | C21H26O13 | 179.03, 193.05 | Other glycosides | Coumarin glycosides |
| M5 | 4.62 | 595.1649 | [M + H]^+^ | -2.35 | Nictoflorin | C27H30O15 | 287.05, 449.10, 471.09 | Flavonoid glycosides | Flavonoid O-glycosides |
| M6 | 4.74 | 729.1642 | [M + Na]^+^ | -0.14 | Kaempferol 3-[2''',3''',5'''-triacetyl-alpha-L-arabinofuranosyl-(1->6)-glucoside | C32H34O18 | 287.05, 471.10 | Flavonoid glycosides | Flavonoid O-glycosides |
| M7 | 4.78 | 713.1578 | [M + H]^+^ | 1.82 | Quercetin 3-O-(6"-malonyl-glucoside) 7-O-glucoside | C30H32O20 | 303.05, 487.08, 573.08 | Flavonoid glycosides | Flavonoid O-glycosides |
| M8 | 4.90 | 611.1651 | [M + H]^+^ | 6.38 | Panasenoside | C27H30O16 | 271.06, 287.05, 433.12 | Flavonoid glycosides | Flavonoid O-glycosides |
| M9 | 5.12 | 705.2348 | [M + Na]^+^ | -3.26 | Scrolepidoside | C32H42O16 | 405.17, 543,19 | Other glycosides | Iridoid glycosides |
| M10 | 5.33 | 863.2257 | [M + Na]^+^ | -6.83 | (+)-alpha-Viniferin 13b-O-beta-glucopyranoside | C48H40O14 | 573.18, 587.19, 629.21, 735.25 | Flavonoid glycosides | arylbenzofuran flavonoids |
| M11 | 5.49 | 713.1523 | [M + H]^+^ | -5.89 | Quercetin 3-O-(6"-malonyl-glucoside) 7-O-glucoside | C30H32O20 | 303.05, 541.15, 563.14 | Flavonoid glycosides | Benzofuran glycosides |
| M12 | 5.55 | 765.2549 | [M + Na]^+^ | -4.31 | Liriodendrin | C34H46O18 | 439.16, 603.20 | Other glycosides | Lignan glycosides |
| M13 | 5.64 | 793.2134 | [M + Na]^+^ | -4.16 | Kaempferol 4'-methyl ether 3-(2Glc-glucosylrutinoside) | C34H42O20 | 301.07, 463.12, 631.17 | Flavonoid glycosides | Flavonoid O-glycosides |
| M14 | 5.83 | 581.1563 | [M + H]^+^ | 9.81 | Rustoside | C26H28O15 | 271.06, 287.05, 471.09 | Flavonoid glycosides | Flavonoid O-glycosides |
| M15 | 5.86 | 697.1616 | [M + H]^+^ | 0 | Kaempferol 3-(6''-malonylglucoside)-7-glucoside | C30H32O19 | 287.05, 449.11, 535.10 | Flavonoid glycosides | Flavonoid O-glycosides |
| M16 | 5.98 | 681.1705 | [M + H]^+^ | 5.58 | Kaempferol 3-(6G-malonylneohesperidoside) | C30H32O18 | 287.05, 617.15 | Flavonoid glycosides | Flavonoid O-glycosides |
| M17 | 6.26 | 393.0808 | [M + H]^+^ | -3.56 | 5,7,3',4',5'-Pentahydroxy-3,6,8-trimethoxyflavone | C18H16O10 | 317.07, 333.05 | Flavonoids | O-methylated flavonoids |
| M18 | 6.30 | 697.1616 | [M + H]^+^ | 0 | Kaempferol 3-(6''-malonylglucoside)-7-glucoside | C30H32O19 | 287.05, 449.10 | Flavonoid glycosides | Flavonoid O-glycosides |
| M19 | 6.67 | 579.17 | [M + H]^+^ | -2.42 | Rhoifolin | C27H30O14 | 163.04, 271.06, 377.08 | Flavonoid glycosides | Flavonoid O-glycosides |
| M20 | 6.73 | 623.1002 | [M + Na]^+^ | -1.77 | Quercetin 3-(2''-galloylrhamnoside) | C28H24O15 | 163.04, 271.06, 519.11 | Flavonoid glycosides | Flavonoid O-glycosides |
| M21 | 6.95 | 433.1143 | [M + H]^+^ | 1.85 | Apigenin-7-O-glucoside | C21H20O10 | 153.02, 271.06 | Flavonoid glycosides | Flavonoid O-glycosides |
| M22 | 6.98 | 447.095 | [M + H]^+^ | 5.14 | Baicalin | C21H18O11 | 153.02, 271.06 | Flavonoid glycosides | Flavonoid O-glucuronides |
| M23 | 7.04 | 609.1787 | [M + H]^+^ | -5.25 | Diosmin | C28H32O15 | 153.02, 287.05, 301.07, 463.12 | Flavonoid glycosides | Flavonoid O-glycosides |
| M24 | 7.10 | 517.1321 | [M + H]^+^ | -4.83 | Isochlorogenic acid A | C25H24O12 | 135.04, 163.04, 377.08 | Others | Quinic acids and derivatives |
| M25 | 7.14 | 723.1816 | [M + H]^+^ | 5.95 | Quercetin 3-(2''',3''',5'''-triacetyl-alpha-L-arabinofuranosyl)(1->6)-glucoside | C32H34O19 | 163.04, 303.05 | Flavonoid glycosides | Flavonoid O-glycosides |
| M26 | 7.29 | 505.1001 | [M + H]^+^ | 3.76 | Luteolin 3'-(3''-acetylglucuronide) | C23H20O13 | 163.04, 285.08, 287.05 | Flavonoid glycosides | Flavonoid O-glucuronides |
| M27 | 7.35 | 463.1233 | [M + H]^+^ | -1.51 | Kaempferide 3-galactoside | C22H22O11 | 286.05, 301.07 | Flavonoid glycosides | Flavonoid O-glycosides |
| M28 | 7.38 | 477.1024 | [M + H]^+^ | -1.89 | Diosmetin 7-O-beta-D-glucuronopyranoside | C22H20O12 | 286.05, 301.07 | Flavonoid glycosides | Flavonoid O-glucuronides |
| M29 | 7.73 | 531.1479 | [M + H]^+^ | -4.52 | Luteolin 7-glucoside-4'-(Z-2-methyl-2-butenoate) | C26H26O12 | 163.04, 271.06, 287.05 | Flavonoid glycosides | Flavonoid O-glycosides |
| M30 | 7.85 | 519.1116 | [M + H]^+^ | -4.43 | Malonylgenistin | C24H22O13 | 153.02, 271.06, 293.04 | Flavonoid glycosides | Isoflavonoid O-glycosides |
| M31 | 8.59 | 287.0546 | [M + H]^+^ | -3.48 | Kaempferol | C15H10O6 | 135.04, 153.20, 179.03 | Flavonoids | Flavonols |
| M32 | 8.66 | 581.1859 | [M + Na]^+^ | 2.24 | Macrophylloside D | C25H34O14 | 315.12, 333.13, 477.18, 495.19 | Other glycosides | Benzopyran glycosides |
| M33 | 8.78 | 317.0671 | [M + H]^+^ | 3.15 | 3-methylquercetin | C16H12O7 | 270.05, 285.08 | Flavonoids | Flavonols |
| M34 | 8.81 | 435.1266 | [M + H]^+^ | -5.75 | Prunin | C21H22O10 | 153.02, 271.06 | Flavonoid glycosides | Flavonoid O-glycosides |
| M35 | 8.85 | 347.0756 | [M + H]^+^ | -3.17 | Syringetin | C17H14O8 | 163.04, 287.06 | Flavonoids | Flavonols |
| M36 | 8.97 | 447.1252 | [M + H]^+^ | -8.72 | Sissotrin | C22H22O10 | 153.02, 271.06 | Flavonoid glycosides | Isoflavonoid O-glycosides |
| M37 | 9.00 | 475.1201 | [M + H]^+^ | -8.21 | Apigenin 7-O-(6''-O-acetylglucoside) | C23H22O11 | 271.06, 293.04 | Flavonoid glycosides | Flavonoid O-glycosides |
| M38 | 9.05 | 561.1224 | [M + H]^+^ | -3.56 | Herbacetin 8-(2'',3'',4''-Triacetylxyloside) | C26H24O14 | 271.06, 293.04 | Flavonoid glycosides | Flavonoid O-glycosides |
| M39 | 9.31 | 591.1333 | [M + H]^+^ | -2.88 | 4,5-dihydroxy-2-{[5-hydroxy-2-(3-hydroxy-4-methoxyphenyl)-4-oxo-4H-chromen-7-yl]oxy}-6-(methoxycarbonyl)oxan-3-yl 1-methyl propanedioate | C27H26O15 | 270.05, 285.08, 301.07 | Flavonoid glycosides | Flavonoid O-glucuronides |
| M40 | 9.71 | 271.0593 | [M + H]^+^ | -4.80 | Apigenin | C15H10O5 | 119.05, 127.04, 153.02 | Flavonoids | Flavones |
| M41 | 9.74 | 561.1224 | [M + H]^+^ | -3.56 | Herbacetin 8-(2'',3'',4''-triacetylxyloside) | C26H24O14 | 153.02, 243.07, 271.06 | Flavonoid glycosides | Flavonoid O-glycosides |
| M42 | 9.98 | 303.0879 | [M + H]^+^ | 3.30 | Hesperetin | C16H14O6 | 153.02, 163.04 | Flavonoids | O-methylated flavonoids |
| M43 | 10.02 | 533.1304 | [M + H]^+^ | 1.69 | Biochanin A 7-O-(6-O-malonyl-beta-D-glucoside) | C25H24O13 | 153.02, 285.08, 307.06 | Flavonoid glycosides | Isoflavonoid O-glycosides |
| M44 | 10.45 | 361.094 | [M + H]^+^ | 4.71 | Acerosin | C18H16O8 | 179.09, 300.06 | Flavonoids | O-methylated flavonoids |
| M45 | 10.76 | 635.2023 | [M + H]^+^ | 7.40 | Acacetin 7-(4'''-acetylrutinoside) | C30H34O15 | 285.0750, 373.11 | Flavonoid glycosides | Flavonoid O-glycosides |
| M46 | 11.29 | 575.1392 | [M + H]^+^ | -1.56 | 6,7,3',4'-Tetrahydroxyaurone 6-O-(3'',4'',6''-tri-O-acetylglucoside) | C27H26O14 | 242.06, 285.08, 307.06 | Flavonoid glycosides | Aurone O-glycosides |
| M47 | 11.76 | 587.0857 | [M + H]^+^ | 5.28 | Bartramiaflavone | C30H18O13 | 285.04, 286.05, 302.04 | Others | Chromones |
| M48 | 11.88 | 375.1084 | [M + H]^+^ | 1.07 | Chrysosplenetin | C19H18O8 | 227.06, 359.07 | Flavonoids | O-methylated flavonoids |
| M49 | 11.95 | 345.0962 | [M + H]^+^ | -3.48 | Ayanin | C18H16O7 | 149.06, 284.07 | Flavonoids | O-methylated flavonoids |
| M50 | 12.69 | 375.1084 | [M + H]^+^ | 1.07 | Casticin | C19H18O8 | 327.05, 345.06 | Flavonoids | O-methylated flavonoids |
| M51 | 12.85 | 285.075 | [M + H]^+^ | -4.56 | Acacetin | C16H12O5 | 153.02, 270.05 | Flavonoids | O-methylated flavonoids |
| M52 | 13.16 | 831.2499 | [M + Na]^+^ | 2.77 | 2-(3,4-dihydroxyphenyl)ethyl 4-{2-[2-(3,4-dihydroxyphenyl)ethoxy]-2-oxoethyl}-3-{2-[(3-phenylprop-2-enoyl)oxy]ethylidene}-2-{[3,4,5-trihydroxy-6-(hydroxymethyl)oxan-2-yl]oxy}-3,4-dihydro-2H-pyran-5-carboxylate | C41H44O17 | 179.07, 579.18, 651.21 | Other glycosides | Terpene glycosides |
| M53 | 13.62 | 359.1137 | [M + H]^+^ | 1.67 | Gardenin B | C19H18O7 | 162.07, 211.07, 298.08, 343.08 | Flavonoids | O-methylated flavonoids |
| M54 | 13.81 | 809.2685 | [M + H]^+^ | 3.46 | 2-(3,4-dihydroxyphenyl)ethyl 4-{2-[2-(3,4-dihydroxyphenyl)ethoxy]-2-oxoethyl}-3-{2-[(3-phenylprop-2-enoyl)oxy]ethylidene}-2-{[3,4,5-trihydroxy-6-(hydroxymethyl)oxan-2-yl]oxy}-3,4-dihydro-2H-pyran-5-carboxylate | C41H44O17 | 161.06, 669.22 | Other glycosides | Terpene glycosides |
| M55 | 13.90 | 371.148 | [M + H]^+^ | -4.04 | Dihydrocurcumin | C21H22O6 | 151.08, 177.09 | Others | Curcuminoids |
| M56 | 14.43 | 389.1273 | [M + H]^+^ | 9.51 | Artemetin | C20H20O8 | 165.07, 328.1 | Flavonoids | O-methylated flavonoids |
| M57 | 16.22 | 518.324 | [M + H]^+^ | -1.35 | LysoPC/18:3 | C26H48NO7P | 125.00, 184.07 | Lipids | LPC |
| M58 | 16.45 | 699.3568 | [M + Na]^+^ | 0 | DGMG (18:3) | C33H56O14 | 277.21, 397.14 (-) | Lipids | DGMG |
| M59 | 16.57 | 476.2804 | [M + H]^+^ | 5.67 | 18:3/LysoPE | C23H42NO7P | 184.04, 297.25 | Lipids | LPE |
| M60 | 16.66 | 518.324 | [M + H]^+^ | -1.35 | 18:3/LysoPC | C26H48NO7P | 125.00, 184.07 | Lipids | LPC |
| M61 | 17.38 | 478.2921 | [M + H]^+^ | -2.72 | LysoPE/18:2 | C23H44NO7P | 311.13, 337.28 | Lipids | LPE |
| M62 | 17.47 | 520.3384 | [M + H]^+^ | -3.65 | LysoPC/18:2 | C26H50NO7P | 125.00, 184.07 | Lipids | LPC |
| M63 | 17.53 | 619.2855 | [M + Na]^+^ | -0.65 | LysoPI/18:2 | C27H49O12P | 283.02, 337.27 | Lipids | LPI |
| M64 | 17.81 | 478.2921 | [M + H]^+^ | -2.72 | 18:2/LysoPE | C23H44NO7P | 109.10, 337.27 | Lipids | LPE |
| M65 | 17.90 | 520.3384 | [M + H]^+^ | -3.65 | 18:2/LysoPC | C26H50NO7P | 125.00, 184.07 | Lipids | LPC |
| M66 | 17.84 | 455.2179 | [M + Na]^+^ | 0.88 | LysoPA/18:3 | C21H37O7P | 98.98, 335.27 | Lipids | LPA |
| M67 | 18.52 | 317.2092 | [M + Na]^+^ | -0.32 | 13-OxoODE | C18H30O3 | 221.16, 261.15 | Lipids | Lineolic acids and derivatives |
| M68 | 18.71 | 454.2928 | [M + H]^+^ | -1.32 | LysoPE/16:0 | C21H44NO7P | 282.28, 313.27 | Lipids | LPE |
| M69 | 18.83 | 496.3366 | [M + H]^+^ | -7.45 | 16:0/LysoPC | C24H50NO7P | 125.00, 184.07 | Lipids | LPC |
| M70 | 18.90 | 531.2706 | [M + H]^+^ | -3.20 | LysoPG/20:5 | C26H43O9P | 253.12, 353.18 | Lipids | LPG |
| M71 | 18.93 | 539.3185 | [M + Na]^+^ | -2.04 | MGMG (18:2) | C27H48O9 | 279.23, 474.15 (-) | Lipids | MGMG |
| M72 | 18.94 | 522.3568 | [M + H]^+^ | 1.53 | LysoPC/18:1 | C26H52NO7P | 125.00, 184.07 | Lipids | LPC |
| M73 | 19.09 | 302.2477 | [M + H]^+^ | -2.32 | (E,E,E)-N-(2-Methylproyl)hexadeca-2,6,8-trien-10-ynamide | C20H31NO | 124.08, 152.11 | Lipids | N-acyl amines |
| M74 | 19.60 | 457.2371 | [M + Na]^+^ | 8.75 | LysoPA/18:2 | C21H39O7P | 275.20, 337.27 | Lipids | LPA |
| M75 | 20.41 | 539.3185 | [M + Na]^+^ | -2.04 | MGMG (18:2) | C27H48O9 | 279.23, 474.15 (-) | Lipids | MGMG |
| M76 | 21.03 | 541.3352 | [M + Na]^+^ | -0.18 | 3-O-α-L-rhamnopyranosyl-3-hydroxyundecanoyl-3-hydroxydecanoic acid | C27H50O9 | 487.37, 541.34 | Lipids | Fatty acids and derivatives |
| M77 | 21.31 | 524.3696 | [M + H]^+^ | -3.81 | 18:0/LysoPC | C26H54NO7P | 125.00, 184.07 | Lipids | LPC |
| M78 | 22.19 | 340.263 | [M + H]^+^ | -2.94 | Evocarpine | C23H33NO | 126.09, 224.20, 302.24 | Others | Hydroquinolones |
| M79 | 23.24 | 331.287 | [M + H]^+^ | 6.64 | MG (16:0) | C19H38O4 | 133.09, 177.11 | Lipids | MG |
| M80 | 23.52 | 314.246 | [M + Na]^+^ | 0 | 1-[(2E,4E)-Tetradecadienoyl]Piperidine | C19H33NO | 138.09, 164.10 | Others | N-acylpiperidines |
| M81 | 24.20 | 411.3492 | [M + H]^+^ | 4.38 | MG (22:2) | C25H46O4 | 133.09, 295.27 | Lipids | MG |
| M82 | 24.26 | 593.2795 | [M + H]^+^ | 5.23 | Pheophorbide A | C35H36N4O5 | 505.23, 533.25 | Others | Tetrapyrroles and derivatives |
| M83 | 25.88 | 567.4217 | [M + Na]^+^ | 6.87 | 3Î²-Phenylacetoxyolean- 12-Ene | C38H56O2 | 165.09, 221.15 | Others | Triterpenoids |
| M84 | 26.03 | 359.3154 | [M + H]^+^ | -1.95 | MG (18:0) | C21H42O4 | 89.06, 133.09 | Lipids | MG |
| M85 | 26.22 | 310.312 | [M + H]^+^ | 3.22 | N-Hexadecanoylpyrrolidine | C20H39NO | 126.09, 142.12 | Others | N-acylpyrrolidines |
| M86 | 27.09 | 571.4011 | [M + Na]^+^ | 6.30 | cholesteryl beta-D-glucoside | C33H56O6 | 295.22, 379.29 | Other glycosides | Steroidal glycosides |
| M87 | 27.83 | 778.5404 | [M + H]^+^ | 2.18 | PC (36:6) | C44H76NO8P | 184.07, 595.47 | Lipids | PC |
| M88 | 27.98 | 736.4925 | [M + H]^+^ | 1.09 | PE (36:6) | C41H70NO8P | 367.24, 553.43, 597.5 | Lipids | PE |
| M89 | 28.95 | 780.5571 | [M + H]^+^ | 3.59 | PC (36:5) | C44H78NO8P | 184.07, 597.49 | Lipids | PC |
| M90 | 29.29 | 797.5183 | [M + Na]^+^ | 0.38 | MGDG (36:6) | C45H74O10 | 613.487, 635.46 | Lipids | MGDG |

**Table S2**. Variable importance in projection values and fold changes of differential metabolites.

| No. | WCP *vs.* YCP | | YCP *vs.* GCP | | WCP *vs.* GCP | | WCNP *vs.* YCNP | | YCNP *vs.* GCNP | | WCNP *vs.* GCNP | |
| --- | --- | --- | --- | --- | --- | --- | --- | --- | --- | --- | --- | --- |
|  | VIP | FC | VIP | FC | VIP | FC | VIP | FC | VIP | FC | VIP | FC |
| M1 | \ | \ | 1.39 | 0.57 | 1.38 | 0.42 | 1.3 | 1.6 | \ | \ | \ | \ |
| M2 | \ | \ | 1.37 | 0.44 | 1.34 | 0.42 | \ | \ | \ | \ | \ | \ |
| M3 | 1.34 | 0.53 | \ | \ | 1.3 | 0.46 | \ | \ | \ | \ | \ | \ |
| M4 | \ | \ | \ | \ | \ | \ | 1.28 | 1.69 | \ | \ | \ | \ |
| M5 | 1.38 | 1.5 | \ | \ | \ | \ | \ | \ | \ | \ | 1.39 | 1.81 |
| M6 | 1.39 | 0.49 | 1.35 | 0.52 | 1.35 | 0.26 | \ | \ | 1.35 | 0.54 | 1.36 | 0.33 |
| M7 | \ | \ | \ | \ | \ | \ | \ | \ | \ | \ | 1.33 | 0.5 |
| M8 | 1.34 | 1.51 | \ | \ | 1.34 | 1.94 | \ | \ | \ | \ | \ | \ |
| M9 | \ | \ | \ | \ | \ | \ | 1.25 | 2.14 | \ | \ | \ | \ |
| M10 | \ | \ | \ | \ | \ | \ | \ | \ | \ | \ | 1.29 | 2.2 |
| M11 | \ | \ | \ | \ | \ | \ | 1.26 | 0.55 | \ | \ | \ | \ |
| M12 | \ | \ | \ | \ | \ | \ | 1.32 | 1.64 | \ | \ | 1.4 | 2.41 |
| M13 | \ | \ | \ | \ | 1.35 | 2.2 | \ | \ | \ | \ | \ | \ |
| M14 | 1.38 | 2.15 | \ | \ | 1.31 | 1.88 | \ | \ | \ | \ | \ | \ |
| M15 | \ | \ | \ | \ | \ | \ | 1.28 | 2.2 | \ | \ | \ | \ |
| M16 | \ | \ | 1.38 | 0.58 | \ | \ | \ | \ | \ | \ | \ | \ |
| M17 | \ | \ | \ | \ | \ | \ | \ | \ | \ | \ | 1.36 | 6.76 |
| M18 | 1.33 | 1.53 | 1.43 | 0.52 | \ | \ | \ | \ | \ | \ | \ | \ |
| M19 | \ | \ | \ | \ | \ | \ | 1.31 | 2.79 | \ | \ | \ | \ |
| M20 | \ | \ | 1.28 | 0.49 | 1.27 | 0.31 | \ | \ | \ | \ | \ | \ |
| M21 | \ | \ | \ | \ | \ | \ | 1.33 | 1.94 | \ | \ | \ | \ |
| M22 | \ | \ | \ | \ | \ | \ | \ | \ | 1.35 | 1.9 | \ | \ |
| M23 | \ | \ | \ | \ | 1.34 | 1.88 | \ | \ | \ | \ | \ | \ |
| M24 | 1.26 | 0.59 | \ | \ | \ | \ | \ | \ | \ | \ | \ | \ |
| M25 | 1.32 | 2.7 | \ | \ | \ | \ | \ | \ | \ | \ | \ | \ |
| M26 | 1.31 | 2.8 | 1.26 | 0.59 | \ | \ | \ | \ | \ | \ | \ | \ |
| M27 | \ | \ | \ | \ | 1.34 | 1.66 | \ | \ | \ | \ | \ | \ |
| M28 | \ | \ | \ | \ | 1.3 | 2.7 | \ | \ | \ | \ | \ | \ |
| M29 | \ | \ | \ | \ | \ | \ | 1.31 | 1.61 | \ | \ | 1.36 | 1.74 |
| M30 | \ | \ | \ | \ | \ | \ | 1.31 | 4.19 | 1.25 | 0.23 | \ | \ |
| M31 | \ | \ | 1.41 | 0.37 | 1.3 | 0.44 | \ | \ | 1.35 | 1.66 | 1.4 | 2.1 |
| M32 | 1.35 | 0.56 | \ | \ | 1.33 | 0.58 | \ | \ | \ | \ | \ | \ |
| M33 | \ | \ | \ | \ | 1.37 | 0.61 | \ | \ | \ | \ | \ | \ |
| M34 | 1.36 | 0.5 | \ | \ | \ | \ | \ | \ | \ | \ | \ | \ |
| M35 | \ | \ | \ | \ | \ | \ | \ | \ | 1.45 | 1.92 | 1.32 | 1.7 |
| M36 | \ | \ | \ | \ | \ | \ | 1.29 | 3.7 | \ | \ | \ | \ |
| M37 | \ | \ | \ | \ | 1.26 | 1.66 | 1.33 | 2.87 | \ | \ | \ | \ |
| M38 | \ | \ | \ | \ | \ | \ | 1.29 | 9.75 | \ | \ | \ | \ |
| M39 | 1.34 | 2.22 | \ | \ | 1.26 | 1.73 | \ | \ | \ | \ | \ | \ |
| M40 | \ | \ | 1.4 | 0.37 | 1.3 | 0.36 | 1.26 | 2.1 | \ | \ | \ | \ |
| M41 | \ | \ | \ | \ | \ | \ | 1.29 | 5.5 | \ | \ | \ | \ |
| M42 | 1.4 | 0.53 | \ | \ | 1.39 | 0.57 | \ | \ | \ | \ | \ | \ |
| M43 | \ | \ | \ | \ | \ | \ | 1.32 | 3.47 | \ | \ | \ | \ |
| M44 | 1.4 | 0.48 | \ | \ | 1.39 | 0.44 | \ | \ | \ | \ | \ | \ |
| M45 | 1.39 | 1.8 | \ | \ | 1.37 | 2 | \ | \ | \ | \ | \ | \ |
| M46 | \ | \ | \ | \ | \ | \ | 1.28 | 5.61 | \ | \ | \ | \ |
| M47 | \ | \ | \ | \ | \ | \ | \ | \ | 1.26 | 1.69 | 1.29 | 2.53 |
| M48 | 1.38 | 0.56 | \ | \ | 1.38 | 0.48 | \ | \ | \ | \ | \ | \ |
| M49 | 1.41 | 0.63 | \ | \ | 1.37 | 0.62 | \ | \ | \ | \ | \ | \ |
| M50 | 1.38 | 0.63 | \ | \ | 1.38 | 0.49 | \ | \ | \ | \ | \ | \ |
| M51 | \ | \ | 1.37 | 0.3 | 1.3 | 0.23 | \ | \ | \ | \ | \ | \ |
| M52 | 1.37 | 0.21 | 1.31 | 1.96 | \ | \ | \ | \ | \ | \ | \ | \ |
| M53 | 1.29 | 0.59 | 1.39 | 0.63 | 1.38 | 0.37 | \ | \ | 1.4 | 0.61 | \ | \ |
| M54 | \ | \ | \ | \ | \ | \ | \ | \ | 1.29 | 3.94 | 1.41 | 3.24 |
| M55 | \ | \ | 1.43 | 0.63 | 1.37 | 0.57 | \ | \ | 1.43 | 0.55 | \ | \ |
| M56 | \ | \ | 1.4 | 0.64 | 1.39 | 0.51 | \ | \ | \ | \ | \ | \ |
| M57 | 1.32 | 0.57 | 1.36 | 1.67 | \ | \ | \ | \ | \ | \ | \ | \ |
| M58 | \ | \ | \ | \ | \ | \ | \ | \ | 1.27 | 0.62 | \ | \ |
| M59 | \ | \ | 1.39 | 1.72 | \ | \ | \ | \ | 1.48 | 0.58 | 1.42 | 0.44 |
| M60 | \ | \ | 1.39 | 2.5 | \ | \ | 1.32 | 0.66 | \ | \ | 1.35 | 0.51 |
| M61 | \ | \ | \ | \ | \ | \ | \ | \ | \ | \ | 1.25 | 0.65 |
| M62 | \ | \ | \ | \ | \ | \ | \ | \ | \ | \ | 1.29 | 0.59 |
| M63 | \ | \ | 1.33 | 2.6 | \ | \ | \ | \ | \ | \ | 1.35 | 1.76 |
| M64 | \ | \ | 1.4 | 1.7 | \ | \ | \ | \ | 1.5 | 0.6 | 1.43 | 0.42 |
| M65 | \ | \ | 1.37 | 1.98 | \ | \ | 1.33 | 0.65 | 1.32 | 0.62 | 1.37 | 0.53 |
| M66 | \ | \ | \ | \ | \ | \ | \ | \ | 1.49 | 0.64 | 1.26 | 0.66 |
| M67 | \ | \ | 1.43 | 0.3 | 1.36 | 0.28 | \ | \ | \ | \ | \ | \ |
| M68 | \ | \ | \ | \ | \ | \ | \ | \ | 1.51 | 0.65 | 1.34 | 0.53 |
| M69 | \ | \ | \ | \ | 1.32 | 2.27 | \ | \ | \ | \ | 1.36 | 0.62 |
| M70 | 1.3 | 0.49 | \ | \ | 1.3 | 0.53 | \ | \ | \ | \ | \ | \ |
| M71 | \ | \ | 1.36 | 0.43 | 1.28 | 0.45 | \ | \ | \ | \ | \ | \ |
| M72 | \ | \ | 1.38 | 0.47 | \ | \ | \ | \ | \ | \ | \ | \ |
| M73 | 1.33 | 0.57 | \ | \ | \ | \ | \ | \ | \ | \ | \ | \ |
| M74 | \ | \ | \ | \ | \ | \ | \ | \ | 1.48 | 0.46 | 1.35 | 0.48 |
| M75 | \ | \ | \ | \ | \ | \ | 1.32 | 0.27 | 1.47 | 0.47 | 1.42 | 0.15 |
| M76 | \ | \ | 1.4 | 0.53 | 1.36 | 0.41 | 1.31 | 0.35 | 1.44 | 0.44 | 1.4 | 0.15 |
| M77 | \ | \ | 1.37 | 1.78 | 1.34 | 2.82 | \ | \ | \ | \ | \ | \ |
| M78 | 1.27 | 0.61 | 1.36 | 2.68 | 1.34 | 2.13 | \ | \ | \ | \ | \ | \ |
| M79 | \ | \ | \ | \ | \ | \ | \ | \ | 1.45 | 0.63 | \ | \ |
| M80 | 1.33 | 0.57 | 1.37 | 2.49 | \ | \ | \ | \ | \ | \ | 1.37 | 1.62 |
| M81 | \ | \ | \ | \ | \ | \ | \ | \ | 1.42 | 0.65 | \ | \ |
| M82 | 1.26 | 0.41 | 1.43 | 0.29 | 1.38 | 0.12 | 1.27 | 0.52 | 1.43 | 0.56 | 1.37 | 0.35 |
| M83 | 1.42 | 0.18 | 1.44 | 0.44 | 1.4 | 0.8 | \ | \ | \ | \ | \ | \ |
| M84 | \ | \ | \ | \ | \ | \ | \ | \ | 1.32 | 0.6 | \ | \ |
| M85 | 1.36 | 0.44 | 1.37 | 2.16 | \ | \ | \ | \ | \ | \ | \ | \ |
| M86 | \ | \ | 1.4 | 0.38 | \ | \ | \ | \ | \ | \ | \ | \ |
| M87 | 1.31 | 0.64 | 1.41 | 4.32 | \ | \ | \ | \ | \ | \ | 1.26 | 0.38 |
| M88 | \ | \ | 1.39 | 3.28 | \ | \ | \ | \ | \ | \ | \ | \ |
| M89 | \ | \ | 1.3 | 3.78 | \ | \ | \ | \ | \ | \ | \ | \ |
| M90 | \ | \ | \ | \ | 1.38 | 0.65 | \ | \ | \ | \ | \ | \ |

“FC” and “VIP” represented the fold change between two groups and the variable importance in projection values of metabolites in OPLS-DA, respectively. The symbol ‘\’ indicated that the metabolite was not differential metabolite between the two groups.**Table S3**. Annotation of differentially expressed genes in key pathways.

| No. | ID | Length | KEGG | KEGG pathway |
| --- | --- | --- | --- | --- |
| 1 | Cluster-48251.53 | 3239 | K00630 glycerol-3-phosphate O-acyltransferase [EC:2.3.1.15] \| (RefSeq) ATS1; phospholipid/glycerol acyltransferase family protein (A) | ko00561: Glycerolipid metabolism; ko00564: Glycerophospholipid metabolism; ko01100: Metabolic pathways |
| 2 | Cluster-78731.1 | 1529 | K00655 1-acyl-sn-glycerol-3-phosphate acyltransferase [EC:2.3.1.51] \| (RefSeq) ATS2; Phospholipid/glycerol acyltransferase family protein (A) | ko00561: Glycerolipid metabolism; ko00564: Glycerophospholipid metabolism; ko01100: Metabolic pathways; ko01110: Biosynthesis of secondary metabolites |
| 3 | Cluster-3258.13 | 2203 | K00993 ethanolaminephosphotransferase [EC:2.7.8.1] \| (RefSeq) AAPT1; aminoalcoholphosphotransferase 1 (A) | ko00440: Phosphonate and phosphinate metabolism; ko00564: Glycerophospholipid metabolism; ko00565: Ether lipid metabolism; ko01100: Metabolic pathways; ko01110: Biosynthesis of secondary metabolites |
| 4 | Cluster-3258.14 | 1332 | K00993 ethanolaminephosphotransferase [EC:2.7.8.1] \| (RefSeq) AAPT1; aminoalcoholphosphotransferase 1 (A) | ko00440: Phosphonate and phosphinate metabolism; ko00564: Glycerophospholipid metabolism; ko00565: Ether lipid metabolism; ko01100: Metabolic pathways; ko01110: Biosynthesis of secondary metabolites |
| 5 | Cluster-3258.19 | 1919 | K00993 ethanolaminephosphotransferase [EC:2.7.8.1] \| (RefSeq) AAPT1; aminoalcoholphosphotransferase 1 (A) | ko00440: Phosphonate and phosphinate metabolism; ko00564: Glycerophospholipid metabolism; ko00565: Ether lipid metabolism; ko01100: Metabolic pathways; ko01110: Biosynthesis of secondary metabolites |
| 6 | Cluster-3258.21 | 2005 | K00993 ethanolaminephosphotransferase [EC:2.7.8.1] \| (RefSeq) AAPT1; aminoalcoholphosphotransferase 1 (A) | ko00440: Phosphonate and phosphinate metabolism; ko00564: Glycerophospholipid metabolism; ko00565: Ether lipid metabolism; ko01100: Metabolic pathways; ko01110: Biosynthesis of secondary metabolites |
| 7 | Cluster-3258.23 | 1751 | K00993 ethanolaminephosphotransferase [EC:2.7.8.1] \| (RefSeq) AAPT1; aminoalcoholphosphotransferase 1 (A) | ko00440: Phosphonate and phosphinate metabolism; ko00564: Glycerophospholipid metabolism; ko00565: Ether lipid metabolism; ko01100: Metabolic pathways; ko01110: Biosynthesis of secondary metabolites |
| 8 | Cluster-3258.11 | 2086 | K00993 ethanolaminephosphotransferase [EC:2.7.8.1] \| (RefSeq) AAPT1; aminoalcoholphosphotransferase 1 (A) | ko00440: Phosphonate and phosphinate metabolism; ko00564: Glycerophospholipid metabolism; ko00565: Ether lipid metabolism; ko01100: Metabolic pathways; ko01110: Biosynthesis of secondary metabolites |
| 9 | Cluster-3258.16 | 1968 | K00993 ethanolaminephosphotransferase [EC:2.7.8.1] \| (RefSeq) AAPT1; aminoalcoholphosphotransferase 1 (A) | ko00440: Phosphonate and phosphinate metabolism; ko00564: Glycerophospholipid metabolism; ko00565: Ether lipid metabolism; ko01100: Metabolic pathways; ko01110: Biosynthesis of secondary metabolites |
| 10 | Cluster-3258.15 | 1616 | K00993 ethanolaminephosphotransferase [EC:2.7.8.1] \| (RefSeq) AAPT1; aminoalcoholphosphotransferase 1 (A) | ko00440: Phosphonate and phosphinate metabolism; ko00564: Glycerophospholipid metabolism; ko00565: Ether lipid metabolism; ko01100: Metabolic pathways; ko01110: Biosynthesis of secondary metabolites |
| 11 | Cluster-3258.17 | 943 | K00993 ethanolaminephosphotransferase [EC:2.7.8.1] \| (RefSeq) AAPT1; aminoalcoholphosphotransferase 1 (A) | ko00440: Phosphonate and phosphinate metabolism; ko00564: Glycerophospholipid metabolism; ko00565: Ether lipid metabolism; ko01100: Metabolic pathways; ko01110: Biosynthesis of secondary metabolites |
| 12 | Cluster-3258.18 | 1616 | K00993 ethanolaminephosphotransferase [EC:2.7.8.1] \| (RefSeq) AAPT1; aminoalcoholphosphotransferase 1 (A) | ko00440: Phosphonate and phosphinate metabolism; ko00564: Glycerophospholipid metabolism; ko00565: Ether lipid metabolism; ko01100: Metabolic pathways; ko01110: Biosynthesis of secondary metabolites |
| 13 | Cluster-3258.9 | 1698 | K00993 ethanolaminephosphotransferase [EC:2.7.8.1] \| (RefSeq) AAPT1; aminoalcoholphosphotransferase 1 (A) | ko00440: Phosphonate and phosphinate metabolism; ko00564: Glycerophospholipid metabolism; ko00565: Ether lipid metabolism; ko01100: Metabolic pathways; ko01110: Biosynthesis of secondary metabolites |
| 14 | Cluster-3258.20 | 1359 | K00993 ethanolaminephosphotransferase [EC:2.7.8.1] \| (RefSeq) AAPT2; aminoalcoholphosphotransferase (A) | ko00440: Phosphonate and phosphinate metabolism; ko00564: Glycerophospholipid metabolism; ko00565: Ether lipid metabolism; ko01100: Metabolic pathways; ko01110: Biosynthesis of secondary metabolites |
| 15 | Cluster-88930.6 | 832 | K01047 secretory phospholipase A2 [EC:3.1.1.4] \| (RefSeq) PLA2-ALPHA; Phospholipase A2 family protein (A) | ko00564: Glycerophospholipid metabolism; ko00565: Ether lipid metabolism; ko00590: Arachidonic acid metabolism; ko00591: Linoleic acid metabolism; ko00592: alpha-Linolenic acid metabolism; ko01100: Metabolic pathways; ko01110: Biosynthesis of secondary metabolites |
| 16 | Cluster-45196.3 | 1066 | K01054 acylglycerol lipase [EC:3.1.1.23] \| (RefSeq) alpha/beta-Hydrolases superfamily protein (A) | ko00561: Glycerolipid metabolism; ko01100: Metabolic pathways |
| 17 | Cluster-45196.7 | 1690 | K01054 acylglycerol lipase [EC:3.1.1.23] \| (RefSeq) alpha/beta-Hydrolases superfamily protein (A) | ko00561: Glycerolipid metabolism; ko01100: Metabolic pathways |
| 18 | Cluster-45196.4 | 1905 | K01054 acylglycerol lipase [EC:3.1.1.23] \| (RefSeq) alpha/beta-Hydrolases superfamily protein (A) | ko00561: Glycerolipid metabolism; ko01100: Metabolic pathways |
| 19 | Cluster-45196.6 | 1543 | K01054 acylglycerol lipase [EC:3.1.1.23] \| (RefSeq) alpha/beta-Hydrolases superfamily protein (A) | ko00561: Glycerolipid metabolism; ko01100: Metabolic pathways |
| 20 | Cluster-45196.8 | 1686 | K01054 acylglycerol lipase [EC:3.1.1.23] \| (RefSeq) alpha/beta-Hydrolases superfamily protein (A) | ko00561: Glycerolipid metabolism; ko01100: Metabolic pathways |
| 21 | Cluster-45196.11 | 2057 | K01054 acylglycerol lipase [EC:3.1.1.23] \| (RefSeq) alpha/beta-Hydrolases superfamily protein (A) | ko00561: Glycerolipid metabolism; ko01100: Metabolic pathways |
| 22 | Cluster-45196.0 | 1905 | K01054 acylglycerol lipase [EC:3.1.1.23] \| (RefSeq) alpha/beta-Hydrolases superfamily protein (A) | ko00561: Glycerolipid metabolism; ko01100: Metabolic pathways |
| 23 | Cluster-45196.1 | 1341 | K01054 acylglycerol lipase [EC:3.1.1.23] \| (RefSeq) alpha/beta-Hydrolases superfamily protein (A) | ko00561: Glycerolipid metabolism; ko01100: Metabolic pathways |
| 24 | Cluster-45196.2 | 1070 | K01054 acylglycerol lipase [EC:3.1.1.23] \| (RefSeq) alpha/beta-Hydrolases superfamily protein (A) | ko00561: Glycerolipid metabolism; ko01100: Metabolic pathways |
| 25 | Cluster-45196.10 | 2051 | K01054 acylglycerol lipase [EC:3.1.1.23] \| (RefSeq) alpha/beta-Hydrolases superfamily protein (A) | ko00561: Glycerolipid metabolism; ko01100: Metabolic pathways |
| 26 | Cluster-45196.13 | 2057 | K01054 acylglycerol lipase [EC:3.1.1.23] \| (RefSeq) alpha/beta-Hydrolases superfamily protein (A) | ko00561: Glycerolipid metabolism; ko01100: Metabolic pathways |
| 27 | Cluster-41832.2 | 1458 | K01054 acylglycerol lipase [EC:3.1.1.23] \| (RefSeq) alpha/beta-Hydrolases superfamily protein (A) | ko00561: Glycerolipid metabolism; ko01100: Metabolic pathways |
| 28 | Cluster-41832.1 | 1451 | K01054 acylglycerol lipase [EC:3.1.1.23] \| (RefSeq) alpha/beta-Hydrolases superfamily protein (A) | ko00561: Glycerolipid metabolism; ko01100: Metabolic pathways |
| 29 | Cluster-25347.27 | 1228 | K01054 acylglycerol lipase [EC:3.1.1.23] \| (RefSeq) alpha/beta-Hydrolases superfamily protein (A) | ko00561: Glycerolipid metabolism; ko01100: Metabolic pathways |
| 30 | Cluster-45196.5 | 2599 | K01054 acylglycerol lipase [EC:3.1.1.23] \| (RefSeq) alpha/beta-Hydrolases superfamily protein (A) | ko00561: Glycerolipid metabolism; ko01100: Metabolic pathways |
| 31 | Cluster-79005.0 | 2010 | K01114 phospholipase C [EC:3.1.4.3] \| (RefSeq) NPC1; non-specific phospholipase C1 (A) | ko00562: Inositol phosphate metabolism; ko00564: Glycerophospholipid metabolism; ko00565: Ether lipid metabolism; ko01100: Metabolic pathways; ko01110: Biosynthesis of secondary metabolites |
| 32 | Cluster-89140.3 | 1619 | K01613 phosphatidylserine decarboxylase [EC:4.1.1.65] \| (RefSeq) PSD1; phosphatidylserine decarboxylase 1 (A) | ko00564: Glycerophospholipid metabolism; ko01100: Metabolic pathways; ko01110: Biosynthesis of secondary metabolites |
| 33 | Cluster-15863.1 | 3425 | K01613 phosphatidylserine decarboxylase [EC:4.1.1.65] \| (RefSeq) PSD2; phosphatidylserine decarboxylase 2 (A) | ko00564: Glycerophospholipid metabolism; ko01100: Metabolic pathways; ko01110: Biosynthesis of secondary metabolites |
| 34 | Cluster-15863.5 | 539 | K01613 phosphatidylserine decarboxylase [EC:4.1.1.65] \| (RefSeq) PSD3; phosphatidylserine decarboxylase 3 (A) | ko00564: Glycerophospholipid metabolism; ko01100: Metabolic pathways; ko01110: Biosynthesis of secondary metabolites |
| 35 | Cluster-15863.2 | 3199 | K01613 phosphatidylserine decarboxylase [EC:4.1.1.65] \| (RefSeq) PSD3; phosphatidylserine decarboxylase 3 (A) | ko00564: Glycerophospholipid metabolism; ko01100: Metabolic pathways; ko01110: Biosynthesis of secondary metabolites |
| 36 | Cluster-78706.3 | 1824 | K03715 1,2-diacylglycerol 3-beta-galactosyltransferase [EC:2.4.1.46] \| (RefSeq) MGD2; monogalactosyldiacylglycerol synthase 2 (A) | ko00561: Glycerolipid metabolism; ko01100: Metabolic pathways |
| 37 | Cluster-78706.6 | 1826 | K03715 1,2-diacylglycerol 3-beta-galactosyltransferase [EC:2.4.1.46] \| (RefSeq) MGD2; monogalactosyldiacylglycerol synthase 2 (A) | ko00561: Glycerolipid metabolism; ko01100: Metabolic pathways |
| 38 | Cluster-50928.6 | 1023 | K06130 lysophospholipase II [EC:3.1.1.5] \| (RefSeq) alpha/beta-Hydrolases superfamily protein (A) | ko00564: Glycerophospholipid metabolism |
| 39 | Cluster-59934.0 | 1133 | K06130 lysophospholipase II [EC:3.1.1.5] \| (RefSeq) alpha/beta-Hydrolases superfamily protein (A) | ko00564: Glycerophospholipid metabolism |
| 40 | Cluster-50928.1 | 873 | K06130 lysophospholipase II [EC:3.1.1.5] \| (RefSeq) alpha/beta-Hydrolases superfamily protein (A) | ko00564: Glycerophospholipid metabolism |
| 41 | Cluster-50928.5 | 1019 | K06130 lysophospholipase II [EC:3.1.1.5] \| (RefSeq) alpha/beta-Hydrolases superfamily protein (A) | ko00564: Glycerophospholipid metabolism |
| 42 | Cluster-50928.9 | 1020 | K06130 lysophospholipase II [EC:3.1.1.5] \| (RefSeq) alpha/beta-Hydrolases superfamily protein (A) | ko00564: Glycerophospholipid metabolism |
| 43 | Cluster-65760.4 | 1365 | K06130 lysophospholipase II [EC:3.1.1.5] \| (RefSeq) alpha/beta-Hydrolases superfamily protein (A) | ko00564: Glycerophospholipid metabolism |
| 44 | Cluster-71278.0 | 2151 | K06130 lysophospholipase II [EC:3.1.1.5] \| (RefSeq) alpha/beta-Hydrolases superfamily protein (A) | ko00564: Glycerophospholipid metabolism |
| 45 | Cluster-81616.4 | 1198 | K06130 lysophospholipase II [EC:3.1.1.5] \| (RefSeq) alpha/beta-Hydrolases superfamily protein (A) | ko00564: Glycerophospholipid metabolism |
| 46 | Cluster-81616.3 | 1138 | K06130 lysophospholipase II [EC:3.1.1.5] \| (RefSeq) alpha/beta-Hydrolases superfamily protein (A) | ko00564: Glycerophospholipid metabolism |
| 47 | Cluster-37874.16 | 1311 | K06130 lysophospholipase II [EC:3.1.1.5] \| (RefSeq) alpha/beta-Hydrolases superfamily protein (A) | ko00564: Glycerophospholipid metabolism |
| 48 | Cluster-37874.4 | 1215 | K06130 lysophospholipase II [EC:3.1.1.5] \| (RefSeq) alpha/beta-Hydrolases superfamily protein (A) | ko00564: Glycerophospholipid metabolism |
| 49 | Cluster-71278.2 | 2182 | K06130 lysophospholipase II [EC:3.1.1.5] \| (RefSeq) alpha/beta-Hydrolases superfamily protein (A) | ko00564: Glycerophospholipid metabolism |
| 50 | Cluster-67292.0 | 2460 | K07407 alpha-galactosidase [EC:3.2.1.22] \| (RefSeq) AGAL1; alpha-galactosidase 1 (A) | ko00052: Galactose metabolism; ko00561: Glycerolipid metabolism; ko00600: Sphingolipid metabolism; ko00603: Glycosphingolipid biosynthesis - globo and isoglobo series; ko01100: Metabolic pathways |
| 51 | Cluster-52749.0 | 2000 | K07407 alpha-galactosidase [EC:3.2.1.22] \| (RefSeq) AGAL1; alpha-galactosidase 1 (A) | ko00052: Galactose metabolism; ko00561: Glycerolipid metabolism; ko00600: Sphingolipid metabolism; ko00603: Glycosphingolipid biosynthesis - globo and isoglobo series; ko01100: Metabolic pathways |
| 52 | Cluster-89515.0 | 1813 | K08730 phosphatidylserine synthase 2 [EC:2.7.8.29] \| (RefSeq) PSS1; phosphatidyl serine synthase family protein (A) | ko00564: Glycerophospholipid metabolism; ko01100: Metabolic pathways; ko01110: Biosynthesis of secondary metabolites |
| 53 | Cluster-89515.2 | 1462 | K08730 phosphatidylserine synthase 2 [EC:2.7.8.29] \| (RefSeq) PSS1; phosphatidyl serine synthase family protein (A) | ko00564: Glycerophospholipid metabolism; ko01100: Metabolic pathways; ko01110: Biosynthesis of secondary metabolites |
| 54 | Cluster-89515.5 | 1614 | K08730 phosphatidylserine synthase 2 [EC:2.7.8.29] \| (RefSeq) PSS1; phosphatidyl serine synthase family protein (A) | ko00564: Glycerophospholipid metabolism; ko01100: Metabolic pathways; ko01110: Biosynthesis of secondary metabolites |
| 55 | Cluster-89515.3 | 1143 | K08730 phosphatidylserine synthase 2 [EC:2.7.8.29] \| (RefSeq) PSS1; phosphatidyl serine synthase family protein (A) | ko00564: Glycerophospholipid metabolism; ko01100: Metabolic pathways; ko01110: Biosynthesis of secondary metabolites |
| 56 | Cluster-49975.3 | 2322 | K09480 digalactosyldiacylglycerol synthase [EC:2.4.1.241] \| (RefSeq) DGD2; digalactosyl diacylglycerol deficient 2 (A) | ko00561: Glycerolipid metabolism; ko01100: Metabolic pathways |
| 57 | Cluster-49975.11 | 3885 | K09480 digalactosyldiacylglycerol synthase [EC:2.4.1.241] \| (RefSeq) DGD2; digalactosyl diacylglycerol deficient 2 (A) | ko00561: Glycerolipid metabolism; ko01100: Metabolic pathways |
| 58 | Cluster-83696.7 | 1037 | K13506 glycerol-3-phosphate O-acyltransferase 3/4 [EC:2.3.1.15] \| (RefSeq) GPAT9; glycerol-3-phosphate acyltransferase 9 (A) | ko00561: Glycerolipid metabolism; ko00564: Glycerophospholipid metabolism; ko01100: Metabolic pathways; ko01110: Biosynthesis of secondary metabolites |
| 59 | Cluster-83696.1 | 1539 | K13506 glycerol-3-phosphate O-acyltransferase 3/4 [EC:2.3.1.15] \| (RefSeq) GPAT9; glycerol-3-phosphate acyltransferase 9 (A) | ko00561: Glycerolipid metabolism; ko00564: Glycerophospholipid metabolism; ko01100: Metabolic pathways; ko01110: Biosynthesis of secondary metabolites |
| 60 | Cluster-83696.5 | 1445 | K13506 glycerol-3-phosphate O-acyltransferase 3/4 [EC:2.3.1.15] \| (RefSeq) GPAT9; glycerol-3-phosphate acyltransferase 9 (A) | ko00561: Glycerolipid metabolism; ko00564: Glycerophospholipid metabolism; ko01100: Metabolic pathways; ko01110: Biosynthesis of secondary metabolites |
| 61 | Cluster-72075.1 | 1816 | K13508 glycerol-3-phosphate acyltransferase [EC:2.3.1.15 2.3.1.198] \| (RefSeq) GPAT4; glycerol-3-phosphate acyltransferase 4 (A) | ko00561: Glycerolipid metabolism; ko00564: Glycerophospholipid metabolism; ko01100: Metabolic pathways; ko01110: Biosynthesis of secondary metabolites |
| 62 | Cluster-72075.2 | 902 | K13508 glycerol-3-phosphate acyltransferase [EC:2.3.1.15 2.3.1.198] \| (RefSeq) GPAT4; glycerol-3-phosphate acyltransferase 4 (A) | ko00561: Glycerolipid metabolism; ko00564: Glycerophospholipid metabolism; ko01100: Metabolic pathways; ko01110: Biosynthesis of secondary metabolites |
| 63 | Cluster-81671.2 | 1299 | K13508 glycerol-3-phosphate acyltransferase [EC:2.3.1.15 2.3.1.198] \| (RefSeq) GPAT6; glycerol-3-phosphate acyltransferase 6 (A) | ko00561: Glycerolipid metabolism; ko00564: Glycerophospholipid metabolism; ko01100: Metabolic pathways; ko01110: Biosynthesis of secondary metabolites |
| 64 | Cluster-81671.3 | 1293 | K13508 glycerol-3-phosphate acyltransferase [EC:2.3.1.15 2.3.1.198] \| (RefSeq) GPAT6; glycerol-3-phosphate acyltransferase 6 (A) | ko00561: Glycerolipid metabolism; ko00564: Glycerophospholipid metabolism; ko01100: Metabolic pathways; ko01110: Biosynthesis of secondary metabolites |
| 65 | Cluster-81671.4 | 1788 | K13508 glycerol-3-phosphate acyltransferase [EC:2.3.1.15 2.3.1.198] \| (RefSeq) GPAT6; glycerol-3-phosphate acyltransferase 6 (A) | ko00561: Glycerolipid metabolism; ko00564: Glycerophospholipid metabolism; ko01100: Metabolic pathways; ko01110: Biosynthesis of secondary metabolites |
| 66 | Cluster-81671.5 | 1796 | K13508 glycerol-3-phosphate acyltransferase [EC:2.3.1.15 2.3.1.198] \| (RefSeq) GPAT6; glycerol-3-phosphate acyltransferase 6 (A) | ko00561: Glycerolipid metabolism; ko00564: Glycerophospholipid metabolism; ko01100: Metabolic pathways; ko01110: Biosynthesis of secondary metabolites |
| 67 | Cluster-79242.0 | 1676 | K13508 glycerol-3-phosphate acyltransferase [EC:2.3.1.15 2.3.1.198] \| (RefSeq) GPAT6; glycerol-3-phosphate acyltransferase 6 (A) | ko00561: Glycerolipid metabolism; ko00564: Glycerophospholipid metabolism; ko01100: Metabolic pathways; ko01110: Biosynthesis of secondary metabolites |
| 68 | Cluster-81671.1 | 1792 | K13508 glycerol-3-phosphate acyltransferase [EC:2.3.1.15 2.3.1.198] \| (RefSeq) GPAT6; glycerol-3-phosphate acyltransferase 6 (A) | ko00561: Glycerolipid metabolism; ko00564: Glycerophospholipid metabolism; ko01100: Metabolic pathways; ko01110: Biosynthesis of secondary metabolites |
| 69 | Cluster-72075.3 | 1863 | K13508 glycerol-3-phosphate acyltransferase [EC:2.3.1.15 2.3.1.198] \| (RefSeq) GPAT8; glycerol-3-phosphate acyltransferase 8 (A) | ko00561: Glycerolipid metabolism; ko00564: Glycerophospholipid metabolism; ko01100: Metabolic pathways; ko01110: Biosynthesis of secondary metabolites |
| 70 | Cluster-77205.0 | 1951 | K13510 lysophosphatidylcholine acyltransferase / lyso-PAF acetyltransferase [EC:2.3.1.23 2.3.1.67] \| (RefSeq) calcineurin B subunit-like protein (A) | ko00564: Glycerophospholipid metabolism; ko00565: Ether lipid metabolism; ko01100: Metabolic pathways |
| 71 | Cluster-77205.1 | 1733 | K13510 lysophosphatidylcholine acyltransferase / lyso-PAF acetyltransferase [EC:2.3.1.23 2.3.1.67] \| (RefSeq) calcineurin B subunit-like protein (A) | ko00564: Glycerophospholipid metabolism; ko00565: Ether lipid metabolism; ko01100: Metabolic pathways |
| 72 | Cluster-77205.4 | 2110 | K13510 lysophosphatidylcholine acyltransferase / lyso-PAF acetyltransferase [EC:2.3.1.23 2.3.1.67] \| (RefSeq) calcineurin B subunit-like protein (A) | ko00564: Glycerophospholipid metabolism; ko00565: Ether lipid metabolism; ko01100: Metabolic pathways |
| 73 | Cluster-77205.7 | 710 | K13510 lysophosphatidylcholine acyltransferase / lyso-PAF acetyltransferase [EC:2.3.1.23 2.3.1.67] \| (RefSeq) calcineurin B subunit-like protein (A) | ko00564: Glycerophospholipid metabolism; ko00565: Ether lipid metabolism; ko01100: Metabolic pathways |
| 74 | Cluster-46779.6 | 625 | K13510 lysophosphatidylcholine acyltransferase / lyso-PAF acetyltransferase [EC:2.3.1.23 2.3.1.67] \| (RefSeq) Phospholipid/glycerol acyltransferase family protein (A) | ko00564: Glycerophospholipid metabolism; ko00565: Ether lipid metabolism; ko01100: Metabolic pathways |
| 75 | Cluster-46779.0 | 1027 | K13510 lysophosphatidylcholine acyltransferase / lyso-PAF acetyltransferase [EC:2.3.1.23 2.3.1.67] \| (RefSeq) Phospholipid/glycerol acyltransferase family protein (A) | ko00564: Glycerophospholipid metabolism; ko00565: Ether lipid metabolism; ko01100: Metabolic pathways |
| 76 | Cluster-46779.2 | 1764 | K13510 lysophosphatidylcholine acyltransferase / lyso-PAF acetyltransferase [EC:2.3.1.23 2.3.1.67] \| (RefSeq) Phospholipid/glycerol acyltransferase family protein (A) | ko00564: Glycerophospholipid metabolism; ko00565: Ether lipid metabolism; ko01100: Metabolic pathways |
| 77 | Cluster-46779.5 | 1764 | K13510 lysophosphatidylcholine acyltransferase / lyso-PAF acetyltransferase [EC:2.3.1.23 2.3.1.67] \| (RefSeq) Phospholipid/glycerol acyltransferase family protein (A) | ko00564: Glycerophospholipid metabolism; ko00565: Ether lipid metabolism; ko01100: Metabolic pathways |
| 78 | Cluster-46779.8 | 1658 | K13510 lysophosphatidylcholine acyltransferase / lyso-PAF acetyltransferase [EC:2.3.1.23 2.3.1.67] \| (RefSeq) Phospholipid/glycerol acyltransferase family protein (A) | ko00564: Glycerophospholipid metabolism; ko00565: Ether lipid metabolism; ko01100: Metabolic pathways |
| 79 | Cluster-84333.0 | 1575 | K13513 lysocardiolipin and lysophospholipid acyltransferase [EC:2.3.1.- 2.3.1.51] \| (RefSeq) LPAT5; lysophosphatidyl acyltransferase 5 (A) | ko00561: Glycerolipid metabolism; ko00564: Glycerophospholipid metabolism; ko01100: Metabolic pathways; ko01110: Biosynthesis of secondary metabolites |
| 80 | Cluster-32747.0 | 1908 | K13519 lysophospholipid acyltransferase [EC:2.3.1.51 2.3.1.23 2.3.1.-] \| (RefSeq) LPLAT1; MBOAT (membrane bound O-acyl transferase) family protein (A) | ko00561: Glycerolipid metabolism; ko00564: Glycerophospholipid metabolism; ko00565: Ether lipid metabolism; ko01100: Metabolic pathways; ko01110: Biosynthesis of secondary metabolites |
| 81 | Cluster-72023.0 | 1097 | K13519 lysophospholipid acyltransferase [EC:2.3.1.51 2.3.1.23 2.3.1.-] \| (RefSeq) LPLAT1; MBOAT (membrane bound O-acyl transferase) family protein (A) | ko00561: Glycerolipid metabolism; ko00564: Glycerophospholipid metabolism; ko00565: Ether lipid metabolism; ko01100: Metabolic pathways; ko01110: Biosynthesis of secondary metabolites |
| 82 | Cluster-85723.0 | 1705 | K13523 lysophosphatidic acid acyltransferase / lysophosphatidylinositol acyltransferase [EC:2.3.1.51 2.3.1.-] \| (RefSeq) LPAT2; lysophosphatidyl acyltransferase 2 (A) | ko00561: Glycerolipid metabolism; ko00564: Glycerophospholipid metabolism; ko01100: Metabolic pathways; ko01110: Biosynthesis of secondary metabolites |
| 83 | Cluster-88784.0 | 3112 | K14674 TAG lipase / steryl ester hydrolase / phospholipase A2 / LPA acyltransferase [EC:3.1.1.3 3.1.1.13 3.1.1.4 2.3.1.51] \| (RefSeq) SDP1; Patatin-like phospholipase family protein (A) | ko00100: Steroid biosynthesis; ko00561: Glycerolipid metabolism; ko00564: Glycerophospholipid metabolism; ko00565: Ether lipid metabolism; ko00590: Arachidonic acid metabolism; ko00591: Linoleic acid metabolism; ko00592: alpha-Linolenic acid metabolism; ko01100: Metabolic pathways; ko01110: Biosynthesis of secondary metabolites |
| 84 | Cluster-88784.11 | 2719 | K14674 TAG lipase / steryl ester hydrolase / phospholipase A2 / LPA acyltransferase [EC:3.1.1.3 3.1.1.13 3.1.1.4 2.3.1.51] \| (RefSeq) SDP1; Patatin-like phospholipase family protein (A) | ko00100: Steroid biosynthesis; ko00561: Glycerolipid metabolism; ko00564: Glycerophospholipid metabolism; ko00565: Ether lipid metabolism; ko00590: Arachidonic acid metabolism; ko00591: Linoleic acid metabolism; ko00592: alpha-Linolenic acid metabolism; ko01100: Metabolic pathways; ko01110: Biosynthesis of secondary metabolites |
| 85 | Cluster-76969.10 | 1386 | K16818 phospholipase A1 [EC:3.1.1.32] \| (RefSeq) DAD1; alpha/beta-Hydrolases superfamily protein (A) | ko00564: Glycerophospholipid metabolism; ko00592: alpha-Linolenic acid metabolism; ko01100: Metabolic pathways; ko01110: Biosynthesis of secondary metabolites |
| 86 | Cluster-76969.4 | 1645 | K16818 phospholipase A1 [EC:3.1.1.32] \| (RefSeq) DAD1; alpha/beta-Hydrolases superfamily protein (A) | ko00564: Glycerophospholipid metabolism; ko00592: alpha-Linolenic acid metabolism; ko01100: Metabolic pathways; ko01110: Biosynthesis of secondary metabolites |
| 87 | Cluster-76969.6 | 1645 | K16818 phospholipase A1 [EC:3.1.1.32] \| (RefSeq) DAD1; alpha/beta-Hydrolases superfamily protein (A) | ko00564: Glycerophospholipid metabolism; ko00592: alpha-Linolenic acid metabolism; ko01100: Metabolic pathways; ko01110: Biosynthesis of secondary metabolites |
| 88 | Cluster-76969.7 | 1561 | K16818 phospholipase A1 [EC:3.1.1.32] \| (RefSeq) DAD1; alpha/beta-Hydrolases superfamily protein (A) | ko00564: Glycerophospholipid metabolism; ko00592: alpha-Linolenic acid metabolism; ko01100: Metabolic pathways; ko01110: Biosynthesis of secondary metabolites |
| 89 | Cluster-76969.8 | 1654 | K16818 phospholipase A1 [EC:3.1.1.32] \| (RefSeq) DAD1; alpha/beta-Hydrolases superfamily protein (A) | ko00564: Glycerophospholipid metabolism; ko00592: alpha-Linolenic acid metabolism; ko01100: Metabolic pathways; ko01110: Biosynthesis of secondary metabolites |
| 90 | Cluster-49980.6 | 808 | K16818 phospholipase A1 [EC:3.1.1.32] \| (RefSeq) DAD1; alpha/beta-Hydrolases superfamily protein (A) | ko00564: Glycerophospholipid metabolism; ko00592: alpha-Linolenic acid metabolism; ko01100: Metabolic pathways; ko01110: Biosynthesis of secondary metabolites |
| 91 | Cluster-76969.12 | 1645 | K16818 phospholipase A1 [EC:3.1.1.32] \| (RefSeq) DAD1; alpha/beta-Hydrolases superfamily protein (A) | ko00564: Glycerophospholipid metabolism; ko00592: alpha-Linolenic acid metabolism; ko01100: Metabolic pathways; ko01110: Biosynthesis of secondary metabolites |
| 92 | Cluster-76969.2 | 1633 | K16818 phospholipase A1 [EC:3.1.1.32] \| (RefSeq) DAD1; alpha/beta-Hydrolases superfamily protein (A) | ko00564: Glycerophospholipid metabolism; ko00592: alpha-Linolenic acid metabolism; ko01100: Metabolic pathways; ko01110: Biosynthesis of secondary metabolites |
| 93 | Cluster-58862.0 | 1499 | K16818 phospholipase A1 [EC:3.1.1.32] \| (RefSeq) DAD1; alpha/beta-Hydrolases superfamily protein (A) | ko00564: Glycerophospholipid metabolism; ko00592: alpha-Linolenic acid metabolism; ko01100: Metabolic pathways; ko01110: Biosynthesis of secondary metabolites |
| 94 | Cluster-76969.3 | 1775 | K16818 phospholipase A1 [EC:3.1.1.32] \| (RefSeq) DAD1; alpha/beta-Hydrolases superfamily protein (A) | ko00564: Glycerophospholipid metabolism; ko00592: alpha-Linolenic acid metabolism; ko01100: Metabolic pathways; ko01110: Biosynthesis of secondary metabolites |
| 95 | Cluster-23489.0 | 1411 | K16818 phospholipase A1 [EC:3.1.1.32] \| (RefSeq) DAD1; alpha/beta-Hydrolases superfamily protein (A) | ko00564: Glycerophospholipid metabolism; ko00592: alpha-Linolenic acid metabolism; ko01100: Metabolic pathways; ko01110: Biosynthesis of secondary metabolites |
| 96 | Cluster-76969.0 | 1633 | K16818 phospholipase A1 [EC:3.1.1.32] \| (RefSeq) DAD1; alpha/beta-Hydrolases superfamily protein (A) | ko00564: Glycerophospholipid metabolism; ko00592: alpha-Linolenic acid metabolism; ko01100: Metabolic pathways; ko01110: Biosynthesis of secondary metabolites |
| 97 | Cluster-49980.2 | 1924 | K16818 phospholipase A1 [EC:3.1.1.32] \| (RefSeq) DAD1; alpha/beta-Hydrolases superfamily protein (A) | ko00564: Glycerophospholipid metabolism; ko00592: alpha-Linolenic acid metabolism; ko01100: Metabolic pathways; ko01110: Biosynthesis of secondary metabolites |
| 98 | Cluster-49980.3 | 1927 | K16818 phospholipase A1 [EC:3.1.1.32] \| (RefSeq) DAD1; alpha/beta-Hydrolases superfamily protein (A) | ko00564: Glycerophospholipid metabolism; ko00592: alpha-Linolenic acid metabolism; ko01100: Metabolic pathways; ko01110: Biosynthesis of secondary metabolites |
| 99 | Cluster-49980.7 | 979 | K16818 phospholipase A1 [EC:3.1.1.32] \| (RefSeq) DAD1; alpha/beta-Hydrolases superfamily protein (A) | ko00564: Glycerophospholipid metabolism; ko00592: alpha-Linolenic acid metabolism; ko01100: Metabolic pathways; ko01110: Biosynthesis of secondary metabolites |
| 100 | Cluster-60928.0 | 1565 | K22389 phospholipase A1 [EC:3.1.1.32] \| (RefSeq) LCAT3; lecithin:cholesterol acyltransferase 3 (A) | ko00564: Glycerophospholipid metabolism; ko00592: alpha-Linolenic acid metabolism; ko01100: Metabolic pathways; ko01110: Biosynthesis of secondary metabolites |
